# Designer Self‐Assembling Peptide Hydrogels to Engineer 3D Cell Microenvironments for Cell Constructs Formation and Precise Oncology Remodeling in Ovarian Cancer

**DOI:** 10.1002/advs.201903718

**Published:** 2020-03-20

**Authors:** Zehong Yang, Hongyan Xu, Xiaojun Zhao

**Affiliations:** ^1^ West China School of Basic Medical Sciences and Forensic Medicine Sichuan University Chengdu Sichuan 610041 P. R. China; ^2^ Institute for Nanobiomedical Technology and Membrane Biology West China Hospital Sichuan University Chengdu Sichuan 610041 P. R. China; ^3^ GL Biochem (Shanghai) Ltd. 519 Ziyue Rd. Shanghai 200241 P. R. China; ^4^ Wenzhou Institute University of Chinese Academy of Sciences (Wenzhou Institute of Biomaterials & Engineering) Wenzhou Zhejiang 325001 P. R. China

**Keywords:** cell constructs, designer self‐assembling peptides, hydrogels, ovarian cancers, precise oncology remodeling

## Abstract

Designer self‐assembling peptides form the entangled nanofiber networks in hydrogels by ionic‐complementary self‐assembly. This type of hydrogel has realistic biological and physiochemical properties to serve as biomimetic extracellular matrix (ECM) for biomedical applications. The advantages and benefits are distinct from natural hydrogels and other synthetic or semisynthetic hydrogels. Designer peptides provide diverse alternatives of main building blocks to form various functional nanostructures. The entangled nanofiber networks permit essential compositional complexity and heterogeneity of engineering cell microenvironments in comparison with other hydrogels, which may reconstruct the tumor microenvironments (TMEs) in 3D cell cultures and tissue‐specific modeling in vitro. Either ovarian cancer progression or recurrence and relapse are involved in the multifaceted TMEs in addition to mesothelial cells, fibroblasts, endothelial cells, pericytes, immune cells, adipocytes, and the ECM. Based on the progress in common hydrogel products, this work focuses on the diverse designer self‐assembling peptide hydrogels for instructive cell constructs in tissue‐specific modeling and the precise oncology remodeling for ovarian cancer, which are issued by several research aspects in a 3D context. The advantages and significance of designer peptide hydrogels are discussed, and some common approaches and coming challenges are also addressed in current complex tumor diseases.

## Graphical abstract

Designer self‐assembling peptides are favorable building blocks compared to other hydrogels, which mimic 3D extracellular matrix at the nanometer scale by bioengineering nanotechnology. In 3D cell cultures, various cell constructs are fabricated by this kind of hydrogels in tissue engineering and regenerative medicine. Herein, the precise oncology remodeling is addressed by several main aspects involved in ovarian cancer.

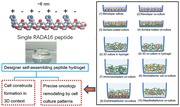

## 1 Introduction

Ex vivo culture of tumor cells from patients has a low culture success rate and a limited proliferative capacity. The most promising strategy expected to improve the success rates is the utility of current nanomedicine involved in the novel biomaterials and advanced hydrogel technologies. Since healthy mouse intestinal stem cells and primary colorectal cancer cells can be propagated in vitro long term,^[^ ^1^ ^]^ there is an urgent need to develop more physiologically relevant, efficient, and robust precise oncology models that closely recapitulate the genetic and morphological heterogeneous composition and mimic the arrangement pattern of cancer cells in the original tumor.^[^ ^2^ ^]^ To our knowledge in biomedical research, hydrogel is the best tool to reconstruct precise oncology models in vitro, especially tumor organoid, which not only recapitulates in vivo biology and microenvironmental factors, but also at large extent allows side‐by‐side comparison to evaluate the translational potential of 3D model systems to the patients.^[^ ^2^, ^3^ ^]^

Generally, hydrogels are mainly composed of hydrophilic polymeric scaffolds that absorb large amounts of water, so the hydrogel matrix better mimics elastic and viscoelastic properties in ECM and microscale topographies of cell matrices, which controls proper cell morphology, directs viable cell behaviors, and drives in vivo fundamental cell–cell or cell–ECM interactions.^[^ ^4^ ^]^ To recapitulate pathophysiological features of human tumors and imitate various aspects of human tumorigenesis in vivo, hydrogels can provide a realistic platform to establish a useful bridge between in vitro assays and in vivo cell microenvironments. In current biomedical applications, there are many kinds of hydrogels to be developed to mimic the biological properties of ECM, such as bulk hydrogels, porous scaffolds, fibrous scaffolds, hydrogel microspheres, hydrogel sandwich systems, microwells, and 3D bioprinted constructs.^[^ ^4^, ^5^ ^]^ Key component in hydrogels is scaffold biomaterial. Pioneered in the 1990s by Zhang and his colleagues performing studies on self‐assembling peptides to serve as ECM for 3D cell culture,^[^ ^6^ ^]^ we have witnessed a concomitant development of biomimetic scaffold biomaterials that mimic the native ECM in vivo at nanoscale and physiologically engineer the cell microenvironment in 3D culture models.^[^ ^4^, ^5^, ^6^, ^7^ ^]^ In natural scaffold biomaterials (e.g., Matrigel, collagen I, silk, and decellularized ECM),^[^ ^7b^ ^]^ their physicochemical properties cannot be readily or independently manufactured or decorated to mimic the ECM of specific disease. In the contrary, synthetic biomaterials can be artificially designed, accurately tuned, and overly modified to mimic the native cell microenvironments and the key factors in ECM components,^[^ ^4^, ^5^ ^]^ For example, a biomimetic type of synthetic hydrogel composed of hyaluronic acid (HA) is rationally designed to mimic the ECM of the diseased lung and reconstruct the complex mechanisms of cell invasion and cell viability in 3D context, where the HA polymer backbone is modified with furan motifs to form tissue‐specific ECMs.^[^ ^7a^ ^]^ A set of PEG‐based synthetic hydrogels are composed of PEG‐macromer containing the enzymatically degradable peptide sequence, GGGPQGIWGQGK, with varying concentrations of the integrin ligating peptide, RGDS (0–10 × 10^−3^ m), which may elucidate the influence of these matrix properties (stiffness, degradability, mesh size, adhesivity) and their independent control on the cancer cell's quiescence and dormancy,^[^ ^8^ ^]^ which is completely distinct from the natural type of hydrogels. As a rapidly growing research field in biomaterials, when well‐designed, synthetic hydrogels may be developed to be ideal functional biomaterials to use as 3D cell culture scaffolds and the popularly utilized tools for tissue‐specific mimicry.

Distinct from synthetic polymeric hydrogels, designer self‐assembling peptide hydrogels are an advanced type of synthetic hydrogels, which may integrate functional, mechanical, chemical, and biological cues by an artificially bioinspired manner. Owing to 20 canonical amino acids in peptide sequence, this type of hydrogels can be extended via the length of synthetic amino acids and tethering properties of peptide backbone to incorporate biologically relevant recognition and signal motifs.^[^ ^9^ ^]^ So, this type of peptide hydrogel has the responsive and adaptive requirements of an artificial ECM for mimicking the native cell microenvironment in vivo. The entangled nanofiber networks in hydrogels confer greatly similar characteristics to the native ECM components in shape, size, and porosity. Due to specifically tailorable biophysical and biomechanical features, it firmly represents advanced synthetic hydrogels capable of providing a tissue‐like but completely synthetic ECM in biomedical applications. Due to the functionality in a user‐directed manner, designer self‐assembling peptide hydrogels can be customized to fabricate the in vivo‐scale adaptable microtissue or cell constructs in basic cancer research, tissue engineering, and regenerative medicine.^[^ ^9b^ ^]^ In cancer nanomedicine, this type of peptide hydrogels is supposed to be a set of versatile matrices for 3D cell culture models, including a wide range of stem cell‐based culture models.^[^ ^9^, ^10^ ^]^

Ovarian cancer is a kind of diseases for a series of molecularly and etiologically distinct occurrences with much stratification of histological or molecular subtypes.^[^ ^11^ ^]^ The pathological and molecular genetics studies suggest that ovarian cancer is characterized by genomic structural variation, with frequent DNA gains and losses, making this cancer an extreme example of a chromosomally unstable (C‐class) malignancy.^[^ ^12^ ^]^ High‐grade serous ovarian cancer (HGSOC) is of particular interest, as it accounts for most deaths from ovarian cancer, and has shown little improvement in overall survival rate in the last 30 years.^[^ ^13^ ^]^ So, modeling ovarian cancer is immensely challenging, due to the genetic complexity, diverse tumor disease pathology, the rare human tissue cell sources for research, the undifined metastasis mechanisms, and the elusive disease origin. More seriously for clinic treatments, it is unclear whether disease relapse and recurrence result from the expansion of self‐renewing cell populations, a change in the ECM, the emergence of drug‐resistant clones or a combination of these events.^[^ ^14^ ^]^ So, the major efforts are not only to characterize recurrent and end‐stage samples but also to develop the precise experimental models that recapitulate the unique biology involved in ovarian cancer initiation, phenotype dormancy, and multistep tumor progression. In previous research, a comprehensive HGSOC model was developed to reflect the clonal diversity and the acquired resistance mechanisms in disease recurrence and relapse.^[^ ^12a^ ^]^ It is an effective strategy that the precise oncology models corroborate intertumor heterogeneity to identify the key genes closely associated with clinical response.^[^ ^15^ ^]^

In this review, we compare natural and synthetic hydrogels currently available. Specially, designer self‐assembling peptide hydrogels are served as the cell culture scaffolds in 3D cell culture models in vitro. We highlight the pivotal role of designer self‐assembling peptide hydrogels to engineer the TMEs in basic cancer research and provide important insights into the precise oncology remodeling of ovarian cancer. These prospects are involved in cancer cell behaviors, exosome and acquired chemoresistance, cell–cell cocultures and cell–ECM interactions, and tumor spheroids formation. Toward engineering 3D cell microenvironment, the aim in this article is to describe these perspectives in these aspects and to inspire researchers to explore designer peptide hydrogels in cancer nanomedicine and precise oncology remodeling in vitro.

## 2 Molecular Self‐Assembly in Designer Peptides and Current Status

In supramolecular chemistry, molecular self‐assembly is a popular and highly efficient strategy to form a large and well‐organized structure to present compositional complexity and achieve most of the functionality for organisms.^[^ ^16^ ^]^ In thermodynamics, molecular self‐assembly is spontaneously motivated by main building blocks in a free system at a global free energy minimum. Among the natural building blocks available, peptides and proteins perform biologically various functionality in body and require high biocompatibility within organisms, due to their initial molecular building blocks to be amino acid residues.^[^ ^17^ ^]^ Accompanied with the decrease of synthetic peptide costs and recent advances in advanced hydrogel techniques,^[^ ^4^, ^18^ ^]^ short designer peptides served as main building blocks in hydrogels are an increasingly popular type of biomaterials.^[^ ^19^ ^]^ Thanks to flexible adaptability and efficient bioavailability, designer peptide hydrogels are the most attractive nanomaterials by forming nanofiber networks with high water content. In principle, short designer peptides are self‐assembled molecule‐by‐molecule or atom‐by‐atom to produce the entangled nanofiber networks and form a variety of hydrogels.^[^ ^20^ ^]^ So, designer peptide hydrogels stereotypically belong to “bottom‐up” instead of “top‐down” construction in tissue engineering and regenerative medicine. In physiochemical properties, self‐assembling peptides have enormous potential as supramolecular biomaterials with accurately tunable mechanical properties and self‐assembled hierarchical nanostructures reminiscent of native protein motifs.^[^ ^21^ ^]^ In bioengineering applications, designer self‐assembling peptide hydrogels are of particular interest in stable, flexible, reproducible, enzyme‐responsive, and multiscale assemblies to form artificial functional nanostructures for therapeutic drug delivery, basic cancer research, tissue engineering, and regenerative medicine. Initially, the curiosity‐driven research in a left‐handed Z‐DNA of yeast pushed the molecular design principles to many directions and gradually understood the dynamic peptide self‐assembly behaviors to form well‐defined functional nanomaterial structures,^[^ ^20^, ^22^ ^]^ which adapt to various biomedical requirements for nanofiber networks. Molecular self‐assembly in short designer peptides objectively requires a deep understanding of individual molecular building block, such as higher‐order structure arrangement, dynamical aggregation process, the tunable mechanical stiffness, and hierarchical stratified alignments at nanoscale.^[^ ^23^ ^]^ Specifically, designer self‐assembling peptides composed of 12 to 24 amino acid residues show classical ionic‐complementary self‐assembly properties in water environments.^[^ ^19^, ^24^ ^]^ They represent the most widely used types of self‐assembling peptides. Because of completely artificial design intention in amino acid compositions, this kind of short designer peptides with several amino acid repeats and alternative polar amino acid arrangement are often termed as designer self‐assembling peptides.

**Figure 1:**
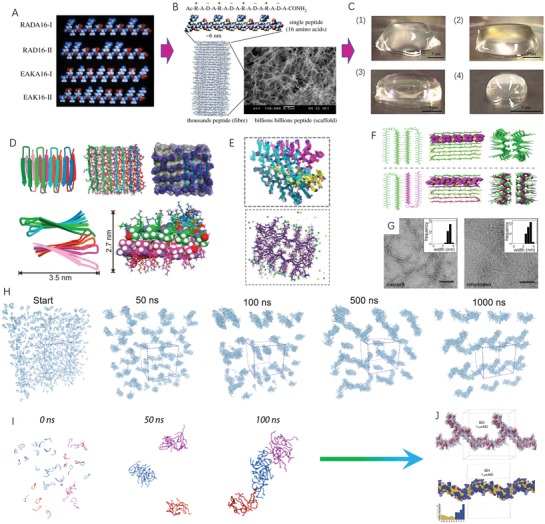
Designer self‐assembling peptide systems and molecular design strategy. A) The molecular models of partial designer peptide moduli. Single molecular building block harbors one hydrophobic side and another hydrophilic side in each modulus, respectively. B) RADA16‐I peptide is at the dimensions with ≈6 nm long, 1.3 nm wide, 0.8 nm thick, and with the charge arrangement ± by four times repeat on the hydrophilic side. Approximately hundreds of thousands or millions of RADA16‐I peptides self‐assemble into a nanofiber architecture depending on the fiber length as revealed by the SEM image. C) Schematic images of RADA16‐I peptide hydrogel at various conditions: (1) 0.5 wt% (pH 7.5), (2) 0.1 wt% (pH 7.5, Tris–HCl), (3) 0.1 wt% (pH 7.5, PBS) before sonication and (4) reassembled RADA16‐I peptide hydrogel after four times of sonication. Reproduced with permission.^[^ ^20^ ^]^ Copyright 2017, The Royal Chemical Society. D) Cartoon, tube, and stick models indicate the molecular relationship between β hairpins in the MAX1 peptide fibrils. E) Superposition of MAX1 peptide structures from 20 equally spaced frames from the final half of the molecular dynamics/Monte Carlo trajectory (upper panel) and final MAX1 fibril structure from molecular dynamics/Monte Carlo calculations (low panel).^[^ ^28a^ ^]^ F) A proposed molecular dynamic model of MAX1/DMAX1 in their coassembled, racemic fibrillar state. ChemDraw figures at top define strand orientation in each assembly. Central images highlight the relative orientation of hairpins within a single monolayer of each fibril type with the valine side chains rendered in CPK (magenta). Bottom images view the racemic and pure enantiomeric fibrils along their long axes.^[^ ^28b^ ^]^ G) Characterization of MAX1 fibrils by TEM and solid‐state NMR. Negatively stained TEM images of nascent MAX1 fibrils (left) and fibrils in a rehydrated hydrogel after lyophilization (right). (Insets) Average nanofibril widths for each sample were ≈3.5 nm (Scale bar = 100 nm). Panels (D–G) are reproduced with permission.^[^ ^28^ ^]^ Copyright 2017, American Chemical Society; Copyright 2015, National Academy of Sciences, USA. H) All snapshots are obtained from a 1 µs restrained atomistic MD simulation of 100 B24 peptides that are initially randomly placed in a water‐filled periodic box. I) Snapshots of B24 peptides, illustrating the preference to assemble into smaller elongated rod‐like clusters that then assemble to larger fibers. J) The supramolecular organization, B24 fiber‐like assembly after 1 µs of restrained atomic MD simulation. Panels (G–I) reproduced with permission.^[^ ^29^ ^]^ Copyright 2019, Wiley‐VCH.

Designer self‐assembling peptides have the alternating charge pattern and hydrophobic and hydrophilic sides on individual molecular building blocks. Generally, the hydrophobic sites are valine, alanine, leucine, isoleucine, and phenylalanine, conversely, the hydrophilic sites are positively charged arginine, lysine, histidine, and negatively charged glutamic acids and aspartic acids, respectively. Based on the hydrophilic surface of molecular building blocks with alternating positively and negatively charged amino acid residues, the classical designer self‐assembling peptides are termed as a type of Lego peptides with ionic‐complementary properties. They have been mainly classified into modulus I, II, III, IV, owing to different charge patterns on building blocks patterning. The alternate charge style in each modulus is following: modulus I, − + − + − + − +; modulus II, − − + + − − + +; modulus III, − − − + + +; and modulus IV, − − − − + + + +, which are alternated by 1, 2, 3, 4 and so on (**Figure** **1**).^[^ ^25^ ^]^ Designer self‐assembling peptides studied so far have the charge orientation described above and show the reverse charge orientations and amino acid residue patterns that produce different molecular building blocks with the defined molecular self‐assembly behaviors. So, they have well‐defined repeated sequences in main building blocks to undergo highly ordered molecular self‐assembly, which is analogous to the circumstances found in the well‐studied synthetic polymers in supramolecular chemistry. This type of molecular self‐assembly principle has paved the foundation of nanofiber architecture formation in designer self‐assembling peptides, including the entangled nanofiber networks and the hydrogelation process in solution.

Based on molecular design strategies above, one more detailed principle is introduced for molecular self‐assembly in short designer peptides. In hierarchical organization arrangement of designer peptides, the formation of hydrogen bonds between the amide backbone and carboxyl group define the secondary structure of individual molecular building block. The geometric structure arising from torsion and curvature of the peptide backbone is used for the location definition of secondary structures, including α‐helix and β‐sheet.^[^ ^19^, ^26^ ^]^ This principle is extensively accepted in protein biochemistry and plays a pivotal role for peptide nanofiber architecture formation. However, designer self‐assembling peptides pave the way to predict the hydrophobic and charge surfaces of designer peptides that drive molecular self‐assembly to form well‐defined nanofiber architecture in the entangled nanofiber networks in hydrogel. The initial dynamic molecular self‐assembly is addressed by Zhang and colleagues,^[^ ^23^, ^27^ ^]^ which is indicated by panels A–C in Figure 1. To understand the mechanism of gelation, the macroscale morphology of fibrillar network, and the underlying molecular structure of fibrils, Nagy‐Smith and co‐workers utilize solid‐state NMR to develop a full structural model for MAX1 fibrils and characterize molecular conformation, β‐sheet organization, and intersheet interactions on all levels of structure (Figure 1D–G).^[^ ^28^ ^]^ To control stem‐cell behaviors beyond nanoscopic‐to‐macroscopic length scales, Jekhmane and colleagues provide atomic‐scale design strategies and related parameters of self‐assembled peptide scaffold by solid‐state NMR approach,^[^ ^29^ ^]^ such as scaffold‐assembly degree, soft or stiff mechanics, well‐defined homogeneity, which reveals a highly ordered nanofibrillar structure at the atomic scale and permit to improve peptide design parameters for favorable stem‐cell scaffolds in implantable cell constructs (Figure 1H–J). Since designer self‐assembling peptides have very consistent sequence characteristics involved in charge and hydrophobicity on the peptide backbone, molecular self‐assembly‐mediated hydrogelation process is better understood compared to other polymer systems,^[^ ^20^ ^]^ such as lipid, polysaccharide, and other chemical polymer. Just because designer self‐assembling peptides have Lego‐like molecular building blocks, hydrophobic intermolecular interactions and charged residue interactions consisted of main driving forces to maintain the well‐defined nanofiber networks architecture in water environment. So, most of designer self‐assembling peptides are readily soluble in water because their amino acid primary sequences are alternating hydrophilic and hydrophobic regions, where 50% charged residues with distinct polar are accompanied with nonpolar surfaces and periodic repeats of two to four times.^[^ ^20^ ^]^ The self‐assembly or hydrogelation is accelerated by millimolar salt concentration under physiological pH solutions or medium. The interwoven nanofiber networks retain extremely high hydration, greater than 99% in water (1–10 mg mL^−1^, w/v).^[^ ^20^, ^23^, ^30^ ^]^ So, designer self‐assembling peptide hydrogel represents an advanced type of nanofiber hydrogels, which may reconstruct the cell milieu in vitro similar to the ECM components in vivo. Since a rational study of the effect produced for each component added to the scaffold (growth factor, polysaccharide or signaling peptide) can be easily carried out,^[^ ^20^ ^]^ it is a good promise to achieve the next generation biomaterials to preserve the native form of growth factor in all hydrogel volumes.^[^ ^24^, ^31^ ^]^ Concurrently, molecular self‐assemblies in peptides and proteins are moving from modulating cellular functionality in 3D context to the predictive creation of new biomimetic nanomaterials by bioengineering strategies at the molecular or atomic levels.^[^ ^29^, ^32^ ^]^ All in all, based on bottom‐up bioengineering strategies the predictive design and biomimetic capacity of designer self‐assembling peptide hydrogels would enhance the development of more physiological and reliable 3D cell models and help the biomedical industry to develop better molecular or cellular therapy approaches in tissue engineering, regenerative medicine, cancer management, or other biomedical applications.

## 3 Common Hydrogel Products and Biomedical Features

Hydrogels are a type of soft materials with high water content and favorable physicochemical characteristics. A fundamental classification of hydrogels based on the polymeric origin is commonly made, such as natural hydrogels and synthetic or semisynthetic hydrogels (**Table** **1**). Herein, we tend to highlight designer self‐assembling peptide hydrogels, so synthetic peptide hydrogels are listed separately with synthetic polymer hydrogels. In biomedical applications, most common hydrogels are widely supposed to naturally derived hydrogels, such as Matrigel, collagen, fibrin, alginate, hyaluronic acid (HA), silk.^[^ ^21^, ^33^ ^]^ Nowadays, these natural hydrogels are popularly applied to biomedical research by bioengineering approaches and prominently served as cell‐based assays in preclinical drug developments, biomedical implants, microfluidic platforms, 3D cell cultures.^[^ ^34^ ^]^ Especially for 3D cell culture systems, Matrigel and collagen I are the gold standards with widespread use in assays and in models in comparison with other types of hydrogels,^[^ ^35^ ^]^ owing to more physiologically relevant capacity.

**Table 1:** Naturally derived and synthetic or semisynthetic hydrogels in biomedical applications

Primary types	Cell scaffolds	Characteristics and advantages	Disadvantages and limitations	Refs.
Natural hydrogels	Matrigel	Collection of collagen, laminin, enactin Multiple growth factors Bioactive sites for cell recognition Good mimic of in vivo cellular conditions Cell phenotype study 3D microenvironment; cytocompatibility; Tunable physical properties.	Complex, chemically not well‐defined scaffold Undefined impurities Unknown amount of growth factors High batch‐to‐batch variation.	^[^ ^35^, ^133^ ^]^
	Collagen I Gelfoam hydrogel	Primary extracellular constituent of ECM Rat tail tendon, tendon, and bovine skin Natural hydrogel‐forming proteins Multiple crosslinking methods Similar structure and stiffness to native tissues Enzymatically degradable properties Native instructive cues for cell recognition.	Require acidic solution to dissolve collagen I Batch‐to‐batch variation Limited control over matrix architecture Inability to tailor its composition.	^[^ ^41^, ^132^, ^144^ ^]^
	Alginate	Linear polysaccharide from brown algae Adhesive ligands for cell attachment Easy cell encapsulation and recovery Biodegradable hydrogel Desired mechanical properties and pore sizes Chemically inert support for cell growth.	Limited cell culture periods Variable stability Mechanical strength The limited modification.	^[^ ^45^, ^46^, ^214^ ^]^
	Hyaluronic acid (HA)	Major glycosaminoglycan in tumor's ECM Tunable chemical modification Biological relevance to tissue in vivo Versatile chemical crosslinking available HA ligand for receptor recognition.	HA hydrogel does not provide integrin attachment	^[^ ^21^, ^136^ ^]^
	Silk fibroin hydrogels	High β‐sheet content and shear thinning Strong adhesive properties Adhesives for medical devices or sensors Therapeutic delivery of (stem) cells	Opaque with the formation of nanocrystallite Low elastic behavior and plastic deformation at strains >10%	^[^ ^224^ ^]^
Semisynthetic hydrogel	GelMA hydrogel	Artificial 3D ECM mimics Gelatin, type I collagen, 70–80% of lysine groups Biocompatibility of natural matrices Reproducibility; Synthetic stability and modularity Tunable properties	Cross‐linked by UV by photoinitiator Teflon mold Amenable stiffness Multiple components	^[^ ^50^, ^197^, ^225^ ^]^
synthetic polymer hydrogels	PEG	User‐controlled modifications Premodified versions and various molecular weights Engineering different functional ligands Degrade via passive, proteolytic, or user‐directed modes Precise tunability of architecture and stiffness	Cell‐binding moieties Biochemical cues Inert substrate Limited cell recovery	^[^ ^21^, ^202^, ^226^ ^]^
	PLGA	Reproducible and tunable physicochemical properties Porous biodegradable synthetic scaffolds Control the type and degree of porosity Good cell attachment properties Amenable to large‐scale use.	Cell‐binding sites Protease‐cleavage motifs Inert substrate Limited cell recovery.	^[^ ^132b^ ^]^
Synthetic peptide hydrogels	PuraMatrix hydrogel	Artificial designer peptide hydrogel Defined amino acid composition Stable β‐sheet and nanofiber structure Great design flexibility Tailorable with specific motifs Biological functionality of native ECM.	The mechanical properties Low stiffness Appropriate rheology.	^[^ ^10^, ^132^, ^170^ ^]^
	Biogelx hydrogel	Self‐supporting nanostructural hydrogels Bioinspired low molecular weight hydrogels Short, simple, di‐ or tri‐peptides N‐terminus modified with the aromatic Fmoc Tunable mechanical and chemical properties Decent stiffness and rheology.	Fmoc groups are not normally found in the ECM;	^[^ ^227^ ^]^

Leighton Joseph is father of 3D tissue culture. He developed collagen sponge‐gel matrix culture system (commercial name was Gelfoam matrix) in the 1950s.^[^ ^36^ ^]^ Gelfoam matrix has been used to culture patient‐derived tumor tissue and achieve native tissue architecture^[^ ^37^ ^]^ in many tumor types, including head and neck cancer,^[^ ^38^ ^]^ gastrointestinal cancer,^[^ ^37^ ^]^ prostate cancer,^[^ ^39^ ^]^ ovarian cancer,^[^ ^40^ ^]^ and so on. In clinical usefulness of Gelfoam histoculture,^[^ ^41^ ^]^ all tumor cell types remain viable and maintain the native architecture for at least 10 d. Gelfoam matrix histoculture permits to determine the cell cycle position of invading and noninvading cancer cells. Cancer cells in G0/G1 phase in Gelfoam matrix histoculture migrate more rapidly than cancer cells in S/G2/M phases.^[^ ^42^ ^]^ Tumor tissue‐like structures are observed only in Gelfoam culture that is remarkably different from those cells in monolayer culture or in Matrigel.^[^ ^43^ ^]^ In Gelfoam matrix drug response assay, both drug‐response spectra of human tumors and in nude mice show that either drug resistance or chemosensitivity in Gelfoam matrix culture highly correlate to the in vivo response at ≈90%.^[^ ^37^, ^44^ ^]^ Although Gelfoam matrix culture is extensively used for patient‐derived tissue cultures in vitro, in scientific community, Gelfoam matrix histoculture is not an active cell culture model in regenerative medicine and tissue engineering except of basic cancer research, since it cannot expand to tumor organoids to maintain genetic complexity for long terms and present compositional heterogeneity and limited manufacture in cell types and ECM components.

Alginate and hyaluronic acid (HA) are two notable types of natural hydrogels,^[^ ^21^ ^]^ since they have highly biological relevance, chemical tunability, and easy amenability to cell encapsulation and cell recovery for downstream assays.^[^ ^21^, ^45^ ^]^ In our body, proteins are not reactive with the alginate and HA components. So, alginate and HA hydrogels function as the relative inert ECMs to support the architecture of the tissue growth in vitro in 3D cell culture manner. Openly spoken, the hydrogel matrix networks mimic salient elements of ECMs while they harbor greatly hydrophilic features, chemical modification, and mechanical amenability similar to those of many soft tissues in vivo. Especially, alginate has flexible tunable porosity and well‐controlled bio degradability. Due to no inherent cell adhesion properties, the alginate hydrogel matrix is often chemically modified by integrin cell binding motifs or combined with other bioactive moieties, so that alginate hydrogel may be used to reconstruct 3D organoid tissues model in vitro.^[^ ^46^ ^]^ As to HA, owing to precise chemical decoration, HA is often used to tailor proper mechanical properties of matrix scaffolds in hydrogels. HA hydrogels are designed to model specific cancer cell behaviors and the phenotypic differences between healthy and diseased cells in 3D context.^[^ ^7a^ ^]^ Silk is natural fibrous protein that is produce by spider or *Bombyx mori* Silk proteins may be processed in aqueous solutions into various biomaterials, such as cell scaffolds, films, hydrogels, microcapsules, and micro‐ and nanospheres,^[^ ^47^ ^]^ which become an excellent candidate for biomedical utility by bio‐nanotechnology. So, these natural hydrogels have high biological efficacy in many clinical and preclinical biomedical applications.

Despite the attractive developments in biomedical applications, due to high lot‐to‐lot variability, undefined matrix composition, and limited chemical modification, these natural hydrogels have been subjected to critical limitations in advanced or precise biomedical technologies for translational medicine, such as spatiotemporally controlled ex vivo microtissue models, biological functionalization incorporated by adhesive and degradable motifs, precisely controlling cell morphology, mechanical stiffness modulations, cell‐specific biomimicry or tissue‐specific components incorporated into hydrogel design, complex multiple cell types construct,^[^ ^4^, ^48^ ^]^ since these biomedical technologies harbor the hierarchical stratified microarchitectures in their native state in vivo, which need be reconstructed by nanoscale methodologies. However, the natural hydrogels in themselves are unable to quantify their composition and characterize their cell binding pockets with cell surface receptors at the nanometer scale. Additionally, in lack of the safety, efficacy and technical feasibility, the natural hydrogels have some prominent drawbacks that cannot be avoided in clinical practice and commercial administration approval.

Luckily, synthetic chemistry has produced some inspired derivatives of native proteins.^[^ ^48^, ^49^ ^]^ Recently, a kind of semisynthetic hydrogel, GelMA hydrogel, is prepared to be applied in a broad range of biomedical researches,^[^ ^50^ ^]^ including 3D bioprinting,^[^ ^51^ ^]^ cardiac patch for heart repair,^[^ ^52^ ^]^ specific tumor cell captures,^[^ ^53^ ^]^ stem cell alignment for tendon tissue engineering,^[^ ^54^ ^]^ the treatment of peripheral nerve damage,^[^ ^55^ ^]^ and identification of tumor cell phenotype.^[^ ^56^ ^]^ Due to the similarities in well‐defined morphological, compositional, and mechanical properties and, when properly designed, the similarities in biological features to the ECM, this kind of semisynthetic hydrogel is relatively a realistic kind of natural biomaterials to potentially use as a substitute of the ECM for reconstructive 3D cell models in tissue engineering, regenerative medicine, basic cancer researches, and some other items. So, with the programmable and customizable hydrogel matrix manufacture platforms to design cell‐laden constructs and mimic 3D cell microenvironment in human being's tissues,^[^ ^49^, ^57^ ^]^ synthetic hydrogels have prominent advantages or realistic bioengineering properties to achieve the biomimetic ECM mimics for cell cultures in vitro and other biomedical applications.

Accompanied with the advance of nanomedicine and nanotechnology, a myriad of hydrogel strategies are now being developed to produce the functional nanostructural biomaterials with defined biological, biochemical, and biophysical features,^[^ ^4^, ^58^ ^]^ which is directing to form a great number of new economic products for clinical use. For examples, Purastat hydrogel is recently licensed for clinical hemostatic nanomaterials in endoscopic resection (ER) surgery^[^ ^59^ ^]^ and suture‐line hemostasis in cardiac surgery.^[^ ^60^ ^]^ The surgeons consistently rate Purastat hydrogel highly, due to the transparent nature and convenient manipulation of the suture site. In chemically synthetic RADA16‐I peptide hydrogels, 3D peptide nanofiber networks are formed by efficient molecular self‐assembly of ionic self‐complementary hexadecapeptide in a pattern of four repeats of four amino acid residues,^[^ ^23b^ ^]^ which not only avoid immunogenicity in human clinic applications but also spontaneously and rapidly form the entangled nanofiber networks without chemical cross‐link reactions and additional components, we suppose that it is a type of precise synthetic nanomaterials that the peptide nanofibers with diameter of about 10–20 nm and maximum length of 500 nm are akin to the native ECM iv vivo. Moreover, if incubated in blood serum, designer RADA16‐I peptides can entangle to be highly‐polymerized peptide nanofibers with 20 times diameter size and 10 times length in solution, that are approximately 200–400 nm fiber and 5 µm length, respectively.^[^ ^61^ ^]^ For proper hemostasis applications,^[^ ^62^ ^]^ marketed under the trade name Purastat by 3‐D Matrix Ltd. is currently most clinical success as topical hemostatic agent.

For cell‐based therapy and drug discovery, designer self‐assembling peptide hydrogels are specifically designed or modified for the tissue cell‐biomimetic customization in a user‐directed manner,^[^ ^3^, ^49^ ^]^ which facilitates the preclinical research translating into clinics or bedside applications. Since Zhang group resulted in the commercial product PuraMatrix hydrogel to be applied in 3D cell culture, regenerative medicine and tissue engineering (3DM Inc., Japan, 2011),^[^ ^20^ ^]^ there are so many commercial products available in recent years,^[^ ^10^, ^21^, ^49^ ^]^ such as PuraMatrix (Corning), PGmatrix (PepGel LLC),^[^ ^21^ ^]^ HydroMatrix (Sigma), Biogelx,^[^ ^63^ ^]^ Purastat and Curodont.^[^ ^49^ ^]^ Due to good biocompatibility, presumable biodegradability, flexible adaptability, high bioavailability, and predictive bioactive capability to interact with tissue or cells, designer self‐assembling peptide hydrogels have been developed to form an advanced type of hydrogels for the prominent advantages to deeply study cell–ECM interactions or stem cell fates in 3D cell cultures in vitro.^[^ ^9^, ^64^ ^]^ Furthermore, designer self‐assembling peptide scaffolds may be designed to realize extensive biological functionality of hydrogel matrices, such as the desired physicochemical properties, desirable mechanical stiffness and possible biological cues for cell growth in vitro, including customizing the inherent native interactions of cells with ECM, and its consequent in situ microtissue remodeling.^[^ ^9^, ^64^, ^65^ ^]^ So, choosing designer self‐assembling peptide hydrogel to culture cells for 3D tissue remodeling in vitro, it is possible to better emulate the physiology of their original ECM in specific tissue types. Compared with various natural hydrogels and synthetic polymer hydrogels (Table 1), in designer self‐assembling peptide hydrogels, dipeptides, tripeptides, tetrapeptide, and their many times repeated peptide sequences are exciting hierarchical main building blocks for various subset of hydrogels, which surely represent biological or inspired candidate motifs to realize molecular bioengineering assembly strategy for precise 3D tissue reconstructs in vitro.

**Figure 2:**
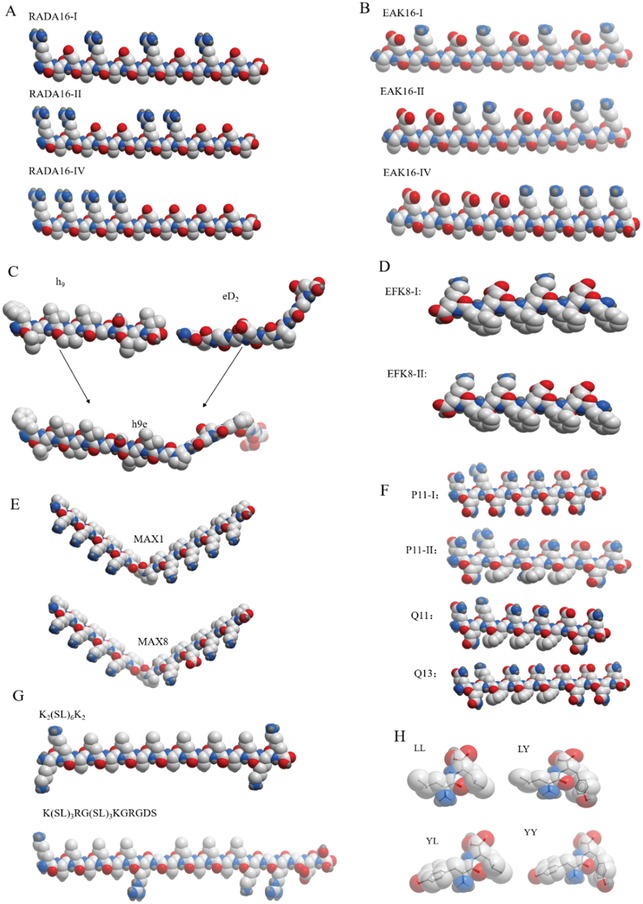
Molecular models of designer self‐assembling peptides currently available in biomedical applications (some other designer peptides are not enclosed here). A) RADA16 modulus peptide shows alternating charged amino acid residues on hydrophilic surface side. The hydrophobic amino acid residues are rationally localized to another β‐sheet side. B) EAK16 modulus peptide shows completely similar molecular building block arrangement with RADA16 modulus peptide except that arginine and aspartic acid residues substitute lysine and glutamate in sequence for salt‐facilitated scaffold formation, since the stable β‐sheet formed is important not only for nanofiber architecture but also for hydrogel formation.^[^ ^215^ ^]^ C) h9e peptide molecular model consists of eD2 and h9 peptide fragments. D) Molecular model of EFK8 peptide (EFK8‐I and EFK8‐II). E) Molecular models of MAX1 and MAX8 peptides, where only one amino acid difference occurs in sequence. F) Molecular models of glutamate‐rich self‐assembling peptides (P11‐I, P11‐II, Q11, and bQ13). G) The multidomain peptides are tethered by the well‐known three amino acid cell adhesion motif (RGD) to promote this variant compatible for cell culture.^[^ ^123b^ ^]^ H) Molecular models of dipeptides (LL, LY, YL, and YY) obtained by atomistic molecular simulations. All short peptide models are produced using the ICM‐Browser software package (MolSoft LLC, San Diego, CA, USA).

Designer self‐assembling peptide hydrogels span over past three decades from serendipitous discovery of the first self‐assembling peptide EAK16‐II in 1990 to a large range of biomedical applications, including cell or drug and antibody carriers,^[^ ^66^ ^]^ stem cell scaffolds,^[^ ^9^, ^67^ ^]^ microtissue formation in vitro,^[^ ^65^ ^]^ and novel peptide detergents or surfactants.^[^ ^23c^ ^]^ The other self‐assembling peptide elements have emerged by a similar molecular design strategy in the past decade years (**Figure** **2**), mainly including glutamine‐rich peptides, β‐hairpin peptides, α‐helix peptides, coiled‐coil peptides, multidomain peptide, and aromatic short peptide derivatives (partially indicated in **Table** **2**).^[^ ^17^ ^]^ Self‐assembling peptide field has nowadays been expanded in a number of directions in nanobiotechnology, such as nanowires,^[^ ^68^ ^]^ nanotubes, nanospheres, nanosheet, and nanoelectronics.^[^ ^19^, ^69^ ^]^ It is a multidisciplinary and complex scientific work to characterize designer peptide building blocks for use as biomimetic nanomaterials. Various reviews cover the basic principles for hydrogel formation by short designer peptide self‐assembly in great details.^[^ ^24^, ^70^ ^]^

**Table 2:** Designer self‐assembling peptide hydrogels to culture cells in bioengineering TMEs for 3D cell cultures

Peptide names	Physiochemical features	Tumor cell types	Description in applications	Refs.
PuraMatrix hydrogel (COCH_3_‐RADARADARADARADA‐CONH_2_)	High‐water content; 3–6 kPa storage modulus; fibril entanglements	A2780, A2780/DDP, SK‐OV‐3, OVCAR‐5 cells; MDA‐MB‐231 and MDA‐MB‐453 stem cells; hepG2 cells; PANC‐1 cells.	Functionalized modification; Easy to isolate cells; Tumor heterogeneity; 3D culture; Drug sensitivity assay; Phenotype presentation	^[^ ^80^, ^142^, ^170^, ^223^ ^]^
EAK16‐II Hydrogel (CONH_3_‐AEAEAKAKAEAEAKAK‐CONH_2_)	The same as RADA16‐I hydrogel	A549; MCF‐7	Easy to isolate cells; High cell viability Drug delivery nanocarrier; Low cytotoxicity; 3D cell culture.	^[^ ^229^ ^]^
H9e peptide hydrogel (FLIVIGSIIGPGGDGPGGD)	Shear‐thinning and easy recovery Self‐assembling hydrogelation.	MCF7 breast cancer cells	Simple cell recovery Tumor‐like cell clusters Superior physiological properties Multiple cell assays; 3D cell cultures.	^[^ ^103^ ^]^
EFK8 hydrogel (EFK8‐I and EFK8‐II) (FEFEFKFK) (EFK8‐SWNT)	Better mechanical strength Disperse carbon nanotubes	NIH‐3T3 cells, A549 cancer cells, MCF7 cancer cells, pluripotent stem cells, MCF10A and MCF10DCIS.	High cell anchorage for attachment, spreading, proliferation and movement The stretched morphology; 3D cell cultures Cell behavior assay Cell–peptide scaffold interactions.	^[^ ^64^, ^207^ ^]^
MAX1 and MAX8 hydrogel [MAX1:VKVKVKVK‐V^D^PPT‐KVKVKVKV‐NH_2_; MAX8:VKVKVKVK‐V^D^PPT‐KVEVKVKV‐NH_2_]	Shear‐thinning behavior Stiffness modulus *G*′ ≈600 Pa Solid hydrogels Rehealing or self‐healing hydrogel	C3H10t1/2 stem cells Osteosarcoma MG63 cells ONS‐76 cells.	Functionalized modification Low‐viscosity gel Unique gel‐cell constructs Homogeneous distribution Controllable hydrogelation Injectable solid hydrogels Drug delivery vehicle.	^[^ ^230^ ^]^
Q_13_ peptide hydrogel Q_13_(Ac‐QQKFQFQFEQEQQAm) Q_11_ (Ac‐QQKFQFQFEQQ‐Am) [P11‐I and P11‐II]	Mildly basic pH Storage moduli 1–10 kPa stiff or rigid hydrogel	C3H10T1/2 stem cells Prostate cancer cells (LNCaP).	3D cell culture Tumor spheroid Chemosensitivity assay Scaffold modification 3D drug testing assay.	^[^ ^114b^ ^]^
Multidomain peptide hydrogel [K_2_(SL)_6_K_2_ and K(SL)_3_RG(SL)_3_KGRGDS]	Ionic or covalent hydrogelation Compatible with ECM Injectable hydrogel.	MOC2‐E6E7, the murine oral cancer cell line; SHED cells	Eightfold slower release rate in collagen hydrogel Easily delivered by syringe Mimic microenvironments in vivo for more complex tissues.	^[^ ^123^, ^231^ ^]^
Fmoc dipeptides [Fmoc‐LL, YL, LY, YY]	MMP‐9 triggered gelation Micelle‐to‐fiber transition.	MDA‐MB‐231‐luc‐D3H2LN cells HEK‐293T cells Osteosarcoma SaOs2 cells; ATDC5 cells.	Enzyme‐responsive properties Tunable properties Site‐specific drug release The fibrillar depots Biocatalytic self‐assembly Variable rigidity and stiffness.	^[^ ^125^, ^232^ ^]^

In designer self‐assembling peptides, the simplest self‐assembly of diphenylalanine (L‐Phe‐L‐Phe) formed hollow peptide nanofibers covered a substantial range from 0 to >300 nm, which is driven to by the hydrophobic phenyl side group with highly stabilized β‐sheet hydrogen bonding via peptide backbones of adjacent molecules.^[^ ^23^, ^71^ ^]^ Additionally, the model octapeptide consists of an alternating sequence of arginine (Arg) and phenylalanine (Phe) residues, namely, [Arg‐Phe]_4_, which forms long unbranched nanofibers with diameters ranging from ≈4 nm up to ≈40 nm and an internal lamellar structure.^[^ ^72^ ^]^ So, these short designer self‐assembling peptides analogously demonstrate the nanofiber structure formation of main building blocks and the hydrogelation formation principle by molecular self‐assembly, which may support the maintenance of the 3D cell culture construct in entangled nanofiber networks with biomimetic biophysical, biomechanical, and structural features over time of cell culture. For the commercial product development, Biogelx Limited is a biomaterials company in UK. A series of ultrashort peptide hydrogels are designed for biomedical or industrial applications. In this type of hydrogel, short, yet simple, di‐ or tri‐peptides modified at the N‐terminus with the aromatic structure, Fmoc, is composed of the hydrogel building blocks and confer Biogelx hydrogels with tunable chemical and mechanical characteristics. Due to their cell‐matched advantages, cell behaviors and functionality can be manipulated by laboratory in the user‐directed manner.^[^ ^63^, ^73^ ^]^ The hydrogelation process is triggered instantly when the Biogelx precursor solution comes into contact with cell culture medium, which is completely analogous to PuraMatrix hydrogel. In basic cancer researches, Biogelx hydrogel is used to generate a large number of microtumors in human cultured for about a month with high viability and drug response testing, which represents a versatile high‐throughput cell model system that can more closely replicate in vivo tumor biology.^[^ ^74^ ^]^ In some other reports,^[^ ^75^ ^]^ this kind of ultrashort peptide hydrogels can be applied to generate hundreds of uniform microtumors within 3–6 d from many types of tumor cells, which are more physiological 3D microtumor models in vitro to investigate how tumor size influences the signaling pathway activation and cancer drug efficacy or amenable to generate a tissue‐specific TME in vitro.

Except of designer peptide hydrogels, poly(ethylene glycol) (PEG) and poly(lactic‐*co*‐glycolic acid) (PLGL) hydrogels are two representatives in synthetic polymer hydrogels, which can be manufactured at large‐scale use and form highly porous scaffolds in a wide range of biomedical applications.^[^ ^21^ ^]^ However, tumor cells cultured in pure synthetic polymer scaffolds can present inconsistent tumorigenicity, metastatic behaviors, resistant phenotypes, and aberrant gene expression patterns.^[^ ^76^ ^]^ So, synthetic polymer scaffolds are often incorporated by other elements such as fibrinogen, chitosan, and hyaluronic acid to produce more robust TME‐mimicking 3D microenvironments. To create synthetic ECM analogues, several nanofibrous peptide amphiphiles (PA) mix with PEG to produce a type of composite hydrogels,^[^ ^77^ ^]^ which mimic essential biochemical and biophysical functionality of the native ECM in vivo in a synergistic manner and form the nanofiber networks architecture in hydrogel. To model breast cancer dormancy, a set of PEG‐based hydrogels are designed by systematic variations in ligand (RGDS) density and crosslink density. Sixteen different hydrogel formulations are used to quantify the temporal response of metastatic breast cancer cells, which extensively analyze the influences of ECM biochemical (ligand (RGDS) density and degradability) and biophysical properties (stiffness and mesh size) on breast cancer cell dormancy, such as viability, apoptotic death, proliferation, metabolic activity, invasiveness, and cell clusters formation over 15 d culture.^[^ ^8^ ^]^ Among these synthetic polymer hydrogels, relatively few are successfully translated into the approved devices and therapeutics, as synthetic polymer hydrogels are simply not made of well‐defined compositions. To achieve an in vivo‐like ECM structure, with the essential microenvironmental cues, is a complex and challenging issue for official approval and commercialization.^[^ ^49^ ^]^ Currently, those that are in clinical use tend to possess the following features: 1) have chemically defined compositions and high bioactivity that are analogous to natural hydrogels; 2) amenable and robust manufacturability with relative ease at minimum cost and with best reproducibility; 3) easy tunability for multiple active components that are well beyond natural hydrogels; 4) completely noncytotoxic effects. Accompanied by the advances in biochemistry, bioengineering techniques, and materials science, synthetic hydrogels will be a product of fundamental work in chemistry, physics, and materials science, and play an pivotal role for understanding of cell–ECM interactions in 3D context,^[^ ^57^ ^]^ especially elaborately depicting the cell binding pockets between hydrogel matrices and cell surface receptors.^[^ ^78^ ^]^ It is possible to create biomimetic studies of cell–ECM interactions and more precise biomechanics in vitro,^[^ ^79^ ^]^ in spite of the inherent complexity in the structure, composition and function of native cell microenvironments in vivo.

Based on these common hydrogel products above, each cell culture system has its own set of advantages and limitations, the best choice often becomes a tradeoff between simplicity of assays in laboratory versus bedside translation of experimental results to clinical usefulness. Designer self‐assembling peptide hydrogels inherently harbor these composite matrix properties to form nanofibrillar architectures with intramolecular folding and intermolecular assembly for hydrogelation process. In designer self‐assembling peptide hydrogels, the canonical amino acid residues serve as basic molecular building blocks of nanofiber scaffold networks, which confer inherent biocompatibility, high hydrophilicity, chemical amenability, and biodegradation in vivo. Because of these prominent advantages, designer self‐assembling peptide hydrogels are proposed to fabricate various 3D ex vivo microtissue models,^[^ ^65^ ^]^ tumor organoids for preclinical drug discovery,^[^ ^80^ ^]^ 3D cell culture constructs for clinical drug repositioning,^[^ ^3^ ^]^ therapeutic drug or cell delivery carrier,^[^ ^66^, ^81^ ^]^ and new generation self‐adjuvant vaccine design.^[^ ^82^ ^]^ Over the past decades, extensive biomedical researches and translational and clinical trials greatly enlarged our understanding of basic cancer research.^[^ ^10^, ^83^ ^]^ A large variety of cancer cell models are available, spanning from monolayer cell culture in petri dishes to 3D cell culture on various designer substrates, or furthermore to achieve precise microtissue remodeling in vitro by bioengineering nanotechnologies and regenerative medicine strategies.

## 4 Diverse Self‐Assembling Peptide Hydrogels and Their Applications

Designer self‐assembling peptide hydrogels are a very active study area. Compared to other types of hydrogels, they are hoped to achieve increased accuracy, exciting diversity, flexible tunability, and physiological relevance to reconstruct 3D cell microenvironments in vitro for basic cancer researches in addition to tissue engineering and regenerative medicine. Accompanied with scientific advances, the synthetic biochemistry techniques allow us to mimic the native ECM in vivo at the user‐directed manner. In this article, by examining previously reported designer self‐assembling peptides by a public software package of ICM‐browser, we list a diverse subset of designer self‐assembling peptides (Figure 2), which indicate the consistent design principle of main building blocks for hydrogel formation. We particularly focus on the significance and advantages of these designer self‐assembling peptide hydrogels and review the state‐of‐the‐art progresses in the bioengineering cell microenvironments for precise 3D cell cultures.

### 4.1 RADA16‐I Peptide Hydrogel

PuraMatrix hydrogel is one representative of synthetic peptide biomaterial family among the most widely used designer self‐assembling peptide hydrogels. The basic molecular building block is a tetrapeptide containing arginine–alanine–aspartate–alanine (RADA) residues with four repetitions. The advantage of this molecular building block, compared with other self‐assembling peptides (Table 2), is its similarity to the RGD (arginine–glycine–aspartic acid residues) tripeptide, a sequence within fibronectin that favors cell attachment or archorage. It has excellent regenerative potential as fillers or scaffolds for a variety of tissue and organ,^[^ ^9b^ ^]^ especially in human neural growth regeneration,^[^ ^67^ ^]^ mesenchymal stem cell transplantation,^[^ ^84^ ^]^ and cardiac cell transplantation therapy for fibrotic tissue remodeling.^[^ ^85^ ^]^ Compared to intramyocardial MSC injection in rat, more cardiac functionality, greater initial retention and survival of donor mesenchymal stem cells (MSCs) are observed in the epicardium with the instantly‐produced PuraMatrix hydrogel incorporating MSCs (epicardial PM‐MSC therapy), by which a group of tissue repair‐related genes are upregulated. Based on the molecular self‐assembly in PuraMatrix hydrogel, the peptide motif QHREDGS derived from angiopoeitin‐1 is tethered to RADA16‐I peptide.^[^ ^84^ ^]^ When carried the MSCs in hydrogel and transplanted into the border of the infarcted cardiac area, the functionalized PuraMatrix hydrogel increases the proliferation of MSCs and decreases apoptosis of MSCs and in situ promote angiogenesis and paracrine by the secretion of IGF‐1 and HGF in rat models. Except of topographic benefits and diverse peptide backbones in RADA16‐I peptide and derivatives, many functional motifs are yet tethered to C‐terminus or N‐terminus of peptide backbone to improve molecular building blocks and accurately mimic the ECM features in vivo. To create a type of permissive neural cell microenvironment for axonal regrowth across lesions,^[^ ^86^ ^]^ IKVAV and RGD functional motifs tethered on RADA16‐I peptide induce more axons regeneration and Schwann cells immigration compared with RADA16‐I hydrogel. [KPSS] is the bioactive motif derived from BMP‐7 molecules. When tethered to C‐terminal of RADA16‐I peptide, the hydrogel properly modulates extracellular microenvironment in intervertebral disc.^[^ ^87^ ^]^ RADA‐KPSS hydrogel enhances the proliferation, differentiation, and chemotactic migration of BMSCs that facilitate intervertebral disc regeneration. A neurite outgrowth peptide IKVAV is bound to RADA16‐I peptide and forms a permissive cell microenvironment to support neuron and astrocyte differentiation, which indicates a new mechanism for nerve regeneration in 3D neuron cell culture in vitro.^[^ ^88^ ^]^ Except of the small peptide sequences described above, IKVAV and YIGSR from laminin are also tethered to C‐terminus of RADA16‐I peptide to prepare the cell differentiation‐triggered matrix scaffold and served as tailor‐made biomimetic cell culture microenvironment.^[^ ^89^ ^]^ Furthermore, a longer laminin motif (CQAASIKVAV (CQIK)) bound with two glycine spacer is tethered to RADA16‐I peptide to support neural differentiation of human endometrial‐derived stromal cells and motor neuron recovery in spinal cord injury.^[^ ^90^ ^]^ Owing to the peptide backbone design, despite the bioactive motif decoration, RADA16‐I peptide hydrogels yet have predominant β‐sheet structure and form the nanofibrous entangled networks in hydrogel. Two longer functional motifs PRGDSGYRGDS and KLTWQELYQLKYKGI bound to RADA16‐I peptide still do not change nanofibrous networks of RADA16‐I peptide in hydrogel and contrarily form a uniform and interwoven long nanofiber architecture with extrusion of functional motifs from the nanofiber surface,^[^ ^91^ ^]^ which not only provide the more optimal bioengineering cell microenvironments for endothelial cell migration and sprouting in vitro, but extensively promote the vessel lumen formation. So, RADA16‐I peptide hydrogels show a broad range of potential in mimicking cell microenvironments in 3D cell models or cell‐based regenerative and reparative therapies.

### 4.2 EAK16‐II Peptide Hydrogel

EAK16‐II peptide is another modulus by two (AEAEAKAK) repetitions in designer self‐assembling peptide system except that replacing aspartate and arginine in RADA16‐I peptide with glutamate and lysine residues. Chen and co‐workers identify the effects of a variety of factors on the peptide self‐assembly mechanism, such as peptide concentration,^[^ ^92^ ^]^ amino acid sequence,^[^ ^93^ ^]^ pH values,^[^ ^93^ ^]^ the medium composition,^[^ ^94^ ^]^ and ionic strength.^[^ ^92^ ^]^ The pH‐dependent self‐assembly behavior of EAK16‐II peptide is elucidated by all‐atom molecular dynamics simulations.^[^ ^95^ ^]^ EAK16‐II is a privileged class of peptide building block, which readily self‐assembles into nanofibrils that entangles to form nanofiber networks in hydrogel by ionic‐complementary self‐assembly with self‐sorting mechanism.^[^ ^96^ ^]^ To regenerate the unique 3D cell microenvironment of the thymic stroma, EAK16‐II peptide hydrogel promotes the thymic epithelial cells (TECs) to form 3D cell aggregates.^[^ ^97^ ^]^ Similar to PuraMatrix hydrogel, the functionalized characteristics are reported in EAK16‐II peptide hydrogel as well. EAK16‐II peptide with six histidine residues (His‐tags) can self‐sort or coassemble into stable β‐sheet structures to achieve in situ self‐gelling nanomaterials.^[^ ^96^ ^]^ This functionalized kind of mechanism is used to develop other Fc‐binding peptide modulus, which provides the bioengineering cell microenvironment for primary thymic epithelial cells to form functional thymus organoids and reconstitute T‐cell adaptive immunity.^[^ ^98^ ^]^ To build precise cell microenvironments for specific cell types, EAK16‐II peptide is conjugated by RGD motif, (GRGDSP)_4_K (fibronectin), FRHRNRKGY (h‐vitronectin), IKVAV (laminin), and type 1 insulin‐like growth factor (IGF‐1), respectively.^[^ ^99^ ^]^ As compared with superficial addition in hydrogel, the conjugation of bioactive motifs with EAK16‐II peptide provides the decoration of the whole hydrogel volume rather than only hydrogel surface. These bioengineering hydrogels support the exchange of bioactive factors, oxygen, nutrients, and waste products between cells and their microenvironment. Specific decoration of EAK16‐II peptide promotes different gene expression in neuronal cells and sustains the functional recovery for enteric nerve regeneration. Various functionalized moieties give rise to different molecular building blocks, which allow for a particularly precise control of cell microenvironment cues. So, EAK16‐II peptide represents a new suite of self‐assembling peptide systems to mimic the complexity of cell microenvironments. EAK16‐II peptide hydrogel containing d‐form amino acid residues favors 3D‐cultured liver cancer cells to have high cell viability and low cell apoptosis.^[^ ^100^ ^]^ Since d‐form peptide sequence is more resistant to protease degradation, d‐form peptide hydrogel can provide more intimate and longer resident extracellular microenvironment for 3D cell cultures. As a consequence of regenerative matrix microenvironments for 3D cell cultures,^[^ ^10^ ^]^ this type of peptide hydrogels are inherently bioactive hydrogels with distinct mechanical and viscoelastic properties in rheology.

### 4.3 h9e Peptide Hydrogel

The h9e peptide is initially designed by combining eD_2_ (GPGGDGPGGD) with a transmembrane segment of FLIVIGSII (h9).^[^ ^101^ ^]^ The eD_2_ sequence favors the elasticity in the extremely high tensile strength of spider silk. The h9 sequence has high adhesion shear strength. Neither eD_2_ alone nor hydrophobic moiety in segment (FLIVI) has the capacity to form hydrogels, only (h9e) FLIVI‐GSII‐GPGGDGPGGD forms strong hydrogel except of any other sequence matches, such as h5e, h5SIIe, h5IIVIe, h5PPDe, L5GSIIe, and h5GSIIK10.^[^ ^101b^ ^]^ This type of hydrogel is shear‐thinning, thermal reversible, water content greater than 99.5%, and 100% cell recovery within 1 min. Moreover, h9e peptide hydrogel has a similar ability to induce an H1N1‐specific IgG1 antibody response compared with an oil‐based commercial adjuvant. In different dimethylsulfoxide (DMSO)/H_2_O solutions, h9e peptide shows nanofiber morphologies and enhances the hydrogelation rate and gel strength as water percentage increases.^[^ ^102^ ^]^ In cancer cell culture researches, h9e peptide hydrogel is a biologically viable scaffold to support MCF7 cancer cells to grow and proliferate by providing in vivo‐like cell microenvironment.^[^ ^103^ ^]^ As to the cell distribution in hydrogel, MCF7 cells are encapsulated homogeneously in the nanofiber matrix during hydrogelation process and form tumor‐like cell clusters. The encapsulated MCF7 cells in 3D culture are able to extrude into the hydrogel and the responses to cisplatin are dose‐ and time‐dependent, which indicates that h9e peptide hydrogel has no apparent negative effect on cell viability and permits the nutrients and drugs to diffuse throughout the hydrogel matrix freely. The cell isolation recovery in h9e peptide hydrogel is safe, effective, and convenient for further biological assay studies, such as western blotting, fluorescence microscopy, and the downstream proteomic analysis. So, h9e peptide hydrogel offers some other interesting properties in 3D cell cultures.

### 4.4 EFK8 Peptide Hydrogel

FEFK (F: phenylalanine, E: glutamic acid, and K: lysine) do not form hydrogels in 0–300 mg mL^−1^ concentration, while two octapeptides FEFKFEFK (EFK8‐I) and FEFEFKFK (EFK8‐II) form the stable hydrogels at low mass concentrations (10 and 15 mg mL^−1^, respectively).^[^ ^104^ ^]^ In peptide sequence, EFK8 peptide nanofibers have better mechanical strength in hydrogel due to stronger hydrophobic interaction of phenylalanine residues, so that EFK16‐II and EFK8 peptides can disperse carbon nanotubes.^[^ ^105^ ^]^ Guilbaud and co‐workers exploit the reverse hydrolytic properties of some enzymes to synthesize self‐assembling peptide hydrogels from a shorter nonself‐assembling peptide precursors.^[^ ^106^ ^]^ They find that the long peptide sequences favor the heterogeneous nanofiber networks in hydrogels, which shows that nanofiber network topology at the micrometer scale directly affects the biophysical properties of these hydrogels. EFK8 peptide hydrogel can form denser nanofiber network regions around the enzymes, which facilitates to engineer the TMEs to truly capture tumor heterogeneity in vivo in 3D tumor models in vitro. EFK8 peptide hydrogel has the tunable compressive modulus that is similar with human lung tissue (<1 kPa) when A549 lung cancer cell spheroid formation in vitro is studied.^[^ ^105^ ^]^ Some A549 cancer cells at the border of tumor spheroids have the stretched morphology and contain less concentrated β‐catenin on the edges and do not have sharp polygonal boundary, which suggests that A549 cells are able to move more easily over the surface. The functionalized EFK‐RGD peptide hydrogel independently controls the matrix stiffness and cell binding site concentration to influence cell spreading and differentiation within the nanofibrous 3D hydrogel matrix.^[^ ^107^ ^]^ To avoid lot to lot variability and compositional or manufactural complexity in probing cell–cell and cell–ECM interactions,^[^ ^64^ ^]^ EFK8 peptide hydrogel combined with fully defined matrix components provides a reliable and reproducible type of 3D cell culture models with independent control of the biochemical and mechanical properties in the extracellular microenvironments in vitro. So, in 3D cell cultures, EFK8 peptide hydrogel may control independently the critical factors: matrix composition and bulk stiffness, which are the key aspects to model the tumor progression from normal breast to breast cancer, including the study of specific cancer cell behaviors.

### 4.5 MAX1 or MAX8 Peptide Hydrogel

MAX1 peptide has a β‐hairpin structure and self‐assembles to be a well‐packed cross‐β‐hairpin architecture by the structural transitions. So, MAX1 peptide hydrogel is one representative in β‐hairpin peptide hydrogels. The hydrogelation is mediated in salt solutions by the desired triggering of intramolecular peptide folding within ≈30 min, which is a unique type of molecular self‐assembly mechanism with concurrent fibril self‐assembly and entanglement into matrix networks compared to other designer peptide hydrogels.^[^ ^33^, ^108^ ^]^ When designed to be MAX8 peptide (replacing the lysine residue at position 15 with glutamic acid residue), that enables swifter folding and faster molecular self‐assembly in monomer within 1 min and forms more rigid hydrogels, this kind of hydrogels is easily injectable, good biocompatible, customizable, and highly responsive to mechanical shear in biomedical applications.^[^ ^109^ ^]^ Both MAX1 and MAX8 form hydrogel matrix network containing a large number of branch points to keep fluidic hydrogel state.^[^ ^110^ ^]^ It is supposed that this very low viscous kind of hydrogel is a good candidate of 3D cell culture scaffolds in vitro for circulating tumor cells in various cancer types,^[^ ^111^ ^]^ although there are few study reports involved in 3D cell culture models in MAX1 or MAX8 peptide hydrogel.

### 4.6 P11 or Q11 Peptide Hydrogel

Initially, Aggeli and colleagues design a kind of self‐assembling peptides with glutamate‐rich residues, including P11‐I (CH_3_CO‐QQRQQQQQEQQ‐NH_2_) and P11‐II (CH_3_CO‐QQRFQWQFEQQ‐NH_2_),^[^ ^112^ ^]^ in which several glutamine residues drive the formation of β‐sheet structure and further form higher‐order nanostructures such as tapes, nanofibers, and fibrils. Recently, P11 peptide is designed to be high aspect‐ratio fibrils that tangle to form hydrogels independently of pH and is developed to be the enamel regeneration product Curodont by creating a local cell microenvironment to enhance enamel mineralization.^[^ ^49^ ^]^ Upon injection in situ, P11 peptides assemble into nanotapes to form the hydrogel matrix that is analogous to the matrix microenvironment necessary for enamel deposition.^[^ ^113^ ^]^ Q11 peptide is derived from glutamate‐rich peptide containing aromatic residues designed by Collier and colleagues.^[^ ^114^ ^]^ In nature, it is the variant of P11 peptide. As Q11 peptide hydrogel is unable to achieve immediate gelation of cell/peptide mixtures in 3D cell culture assay, a modified variant of Q11 peptide, bQ13 is designed to be soluble at mildly basic pH and displays well cytocompatibility amenable to 3D cell culture assay, which considerably improves the viability and growth of prostate cancer cells.^[^ ^114b^ ^]^ Q11 or bQ13 peptide also allows chemical decoration on peptide backbone and fibril elongation,^[^ ^115^ ^]^ Exactly, the termini of Q11 peptide is menable to tether various small chemical moieties or short peptides to mimic the ECM in vivo and delivery immunogenic epitopes to develop next generation vaccine, such as RDGS‐, IKVAV‐, and OVA.^[^ ^116^ ^]^ Beyond the focus in this review, the reader is referred to other reviews for more expansive descriptions in immunological context.^[^ ^49^, ^117^ ^]^ Potentially fruitful future work is greatly attractive in the developments of safe, immunogenic, noninflammatory vaccine products.

### 4.7 Multidomain Peptide Hydrogel

Multidomain peptide (MDP) is amphiphilic with a modular ABA block motif to form β‐sheet structure by dimerizing to protect the nonpolar core.^[^ ^118^ ^]^ A classical MDP motif can self‐assemble into nanofiber networks with the dimensions of 2 × 6 × 120 nm.^[^ ^119^ ^]^ Careful selection of the amino acid residues in the A and B blocks may control nanofiber length and diameter, gelation conditions, and viscoelastic properties in hydrogel. The cell migration and spatial cell spreading may be modulated in 3D cell cultures.^[^ ^120^ ^]^ Interestingly, when aliphatic hydrophobic amino acids in the central core of the peptide backbone are replaced by the aromatic amino acids phenylalanine, tyrosine, and tryptophan, the basic nanofibrous morphology in hydrogel is retained in all cases.^[^ ^121^ ^]^ In principle, it is evident that MDP represents an ideal case of bottom‐up design in tissue engineering and regenerative medicine. Especially, the scaffold degradation rate in vivo varies from as rapidly as 1 week to well over 6 weeks as the MDP nanofibers are degraded to their amino acid components. Functional motif can be displayed at a very high density on the nanofiber's surface to influence cellular behaviors, including new blood vessel formation.^[^ ^122^ ^]^ So far, 29 MDP sequences are previously identified in biomedical fields. These MDP hydrogels currently orient the aligned and multilayered bioactive architecture in 3D tissue or cell constructs to reconstruct the complex stratified tissues in regenerative medicine and tissue engineering.^[^ ^123^ ^]^ Additionally, MDP hydrogels serve as biocompatible and bioactive pulp‐capping materials to induce dentin bridge formation without causing cytotoxic effects, when injected either at the interface of the odontoblasts and the dentin or into the pulp core of mandible slices,^[^ ^124^ ^]^ which is similar with P11 peptide hydrogels to develop as pulp‐capping agents in oral clinical applications.

### 4.8 Fmoc Peptide Hydrogel

Fmoc peptide hydrogels^[^ ^125^ ^]^ have ultrashort peptide building blocks and enzyme‐responsive control potential, that opens up another path to design tailor‐made biocatalytic cell microenvironment in biomedical research and serve as a useful platform to customize tissue‐specific cell culture hydrogels. The dipeptide and tripeptide sequences are the common approaches to show the peptide self‐assembly at the nanometer scale, which is discovered and expanded by Gazit and Ulijn et al. and studied widely to serve as discrete nanostructures in the broad areas.^[^ ^68^, ^126^ ^]^ Various factors can drive gelation formation of these ultrashort peptide sequences, such as amino acids residues, intermolecular interactions in noncovalent forces, the chirality change.^[^ ^127^ ^]^ The exchange of just one amino acid may result in systematic alterations of peptide nanostructures, such as the tunable kinetics, mesh size, and scaffold morphologies at the nanometer scale.^[^ ^128^ ^]^ In biomedical applications, this type of ultrashort peptide self‐assembly and hydrogelation occurs mainly on the cell surface and induce a reduction of the SaOs2 metabolic activity to control cancer cell fate.^[^ ^129^ ^]^ To develop more novel scaffold biomaterials in tissue‐engineered skin, Fmoc peptide hydrogel deposits largely dense ECM networks including fibronectin and collagen I within the tissue site in a 14 d culture period.^[^ ^130^ ^]^ Surprisingly, introducing chemical functionality to Fmoc peptide hydrogel can provide tunable, chemical, and mechanical properties for 3D cell cultures in vitro as well.^[^ ^73^ ^]^ As of today, Fmoc‐Phe‐Phe (FF) dipeptide is still one of the most popularly studied building blocks for hydrogels formation,^[^ ^19b^ ^]^ since it tends to form well biocompatibility and high mechanical stiffness of cell scaffold in some defined biomedical applications. In practice, ultrashort peptides are often produced at low cost, easy bioavailability in vivo and more amenable to molecular dynamics simulations compared with classical designer self‐assembling peptides. So, in the coming years, this type of ultrashort peptide hydrogels will be rapidly developed to be commercial products in many directions.

As described above, designer self‐assembling peptides are a subset of advanced nanomaterials, which are diverse, robust, biological, and convenient in sequence, composition, design, manufacture, manipulation, and transportation by commercial products. As a result of reports described previously, they presumably adapt to the distinctive requirements of synthetic cell scaffolds to reconstruct bioengineering cell microenvironments for 3D cell culture in vitro and microtissue organoid.^[^ ^131^ ^]^ i) Amenable to control hydrogel stiffness around a physiological range; ii) short and nontoxic hydrogelation reactions; iii) biodegradable or labile ionic crosslinks to form the physical networks in hydrogel; and iv) the predictive adhesive sites for cell anchorage. Since 20 canonical amino acids are the initial elements for molecular building blocks in scaffold, using these defined amino acid components, one can have a better control over the quantity of ECM components and their inherent functionality. Due to the amphiphiles in molecular building blocks and possible alternatives to lipids or synthetic polymers, designer self‐assembling peptide hydrogels are attractive for biomedical applications, whereas both Matrigel and collagen I have nonquantified composition, few tunability, xenograft sources, and limited bioavailability.^[^ ^132^ ^]^ In tumor tissue in vivo, the native ECM is a 3D network composed of fiber‐like matrix proteins (e.g., collagen, fibrin, and elastin), that are analogous to designer peptide hydrogel matrix networks in a nanometer scale, so designer peptide nanofiber scaffolds by molecular self‐assembly can better mimic the microscale fibrous networks of the native ECM.^[^ ^133^ ^]^ To develop more physiologically relevant human cancer models, designer self‐assembling peptide hydrogels are premier options for efficient translation of basic cancer research into treatment regimens for patients with cancer.

Although there are many 3D cell culture models reported in designer peptide hydrogels for basic cancer research (Table 2), few molecular‐leveled peptide backbone decorations are identified to remodel tissue‐specific TMEs in vitro involved in specific tissue or cell subtypes, that may be a challenging task in current matrix biology community.^[^ ^64^ ^]^ Due to bioengineering cell microenvironments in designer self‐assembling peptide hydrogels, it is possible to tailor tissue‐specific TMEs in 3D cell culture models in vitro and fine recapitulate critical steps in the metastatic process, such as angiogenesis, intravasation, extravasation, ECM remodeling or adhesion, and cancer cell phenotype dormancy.^[^ ^83^, ^134^ ^]^ Advances in natural or synthetic biomaterials and cancer cell biology have enabled us to develop some interesting, reproducible, robust, and scalable peptide hydrogelation strategies for advanced or precise oncology research, which facilitate us to compare some comprehensive factors, such as the mechanical properties of ECM (stiffness, rigidity, and viscoelasticity), matrix density and space alignment, ECM architecture and spatial topography.^[^ ^134b^ ^]^ Further technical developments would rapidly promote us to control the architecture, mechanics, chemicals and biology of artificial ECM components in vitro in a precise and reproducible way. We suppose that the bioengineering tumor microenvironments in vitro have to tailor cancer cell‐ECM interactions as like two sides of a coin. Generally, it is difficult for us to change selected cancer cell except of genetic modification, so advanced biomaterials in nanomedicine, due to inherent chemical or biological versatility with the composition and the GRAS (generally recognized as safe) status (21CFR72.320) of their amino acid breakdown products, are historically pushed to the center interdisciplinary position, which inspires us to establish the bioengineering tissue cell constructs to better mimic human being's native milieu in vivo in various tissue types.

## 5 Instructive Cell Constructs in Tissue Engineering and Precise Oncology Remodeling in Ovarian Cancer

In our body, life is a complex system, which contains ≈37 trillion cells to make up at least 200 distinct cell types.^[^ ^135^ ^]^ So, cell patterns in specific tissue in vivo and cell type diversity cannot be ignored when fabricating new cell culture models for multicellular constructs, organogenesis, regenerative medicine, and tissue engineering. In scientific community, traditional 2D cell cultures are well established previously and straightforward to pursue cell assays on plastic substrates. But it obscures the critical roles of cell microenvironments in tumorigenesis, tissue morphogenesis, cell development, and organ remodeling. So, the shortcomings of traditional 2D cell culture are widely acknowledged, as this method cannot recapitulate biologically essential processes including cellular morphology, intracellular cytoskeleton, gene expression, cell polarity, inaccurate drug screening, and more others.^[^ ^136^ ^]^ We struggle to approximate the architecture of living tissues experimentally by 3D cell cultures. Much efforts are made to combine cells, scaffolds, and bioactive factors to form instructive cell constructs in a variety of ex vivo cell models by bioengineering nanotechnologies. Herein, based on designer peptide hydrogels in biomedical applications, we mainly discuss the diverse cell constructs for tissue‐specific modeling and precise oncology remodeling strategies for ovarian cancer disease researches.

### 5.1 Instructive Cell Constructs for Tissue‐Specific Modeling

**Figure 3:**
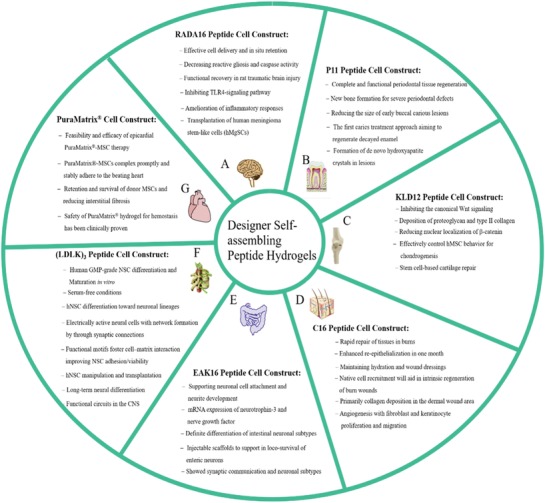
Schematic cell constructs are recently developed for tissue‐specific modeling, which is available or served as already tested tissue remodeling strategies in designer self‐assembling peptide hydrogels. A) Human stem cell‐based cell construct provides alternative options for peripheral nerve regeneration and repair strategies in RADA16‐I hydrogel,^[^ ^65^ ^]^ in addition to the cell construct in EFK‐I hydrogel, which is effectively injectable cell delivery platform for intervertebral disc repair applications.^[^ ^66b^ ^]^ B) P11 peptide hydrogel directly indicates in situ clinical treatment benefit for early buccal carious lesions.^[^ ^216^ ^]^ Other designer peptide hydrogels are the effective cell scaffolds for periodontal regeneration or dental pulp‐derived stem cell transplantation.^[^ ^9b^ ^]^ C) N‐cadherin mimetic peptide nanofiber hydrogel and KLD12 hydrogel are able to enhance cell‐to‐cell interactions by a tissue‐specific modeling strategy to support mesenchymal stem cells (MSC) commitment into the chondrogenic lineage.^[^ ^171^, ^217^ ^]^ D) Arg‐Gly‐Asp‐Ser (RGDS)‐modified peptide amphiphile (PA) hydrogel generate a type of instructive cell construct for the epidermis, neovascularization, and proliferation of fibroblasts.^[^ ^218^ ^]^ E) Functionalized EAK16‐II hydrogels offer the tools to inject or dab the tissue‐specific cell construct onto intestinal areas affected by loss of loconeurons to treat the gastrointestinal disorders caused by the lack of specific neuronal subpopulations.^[^ ^99^ ^]^ F) The hNSC‐seeded cell construct in (LDLK)_3_ hydrogel provides three translational possibilities in synthetic designer peptide scaffolds, GMP‐hNSCs, and serum‐free cultures in nanotechnology.^[^ ^67^ ^]^ G) Instantly produced PuraMatrix hydrogel‐MSC complex is an advanced cell construct for innovative therapy in rat heart failure models,^[^ ^85^ ^]^ since the feasibility and efficacy of Purastat hydrogel achieves the clinical approval in suture line hemostasis.^[^ ^60^ ^]^ Panels (A–G) are reproduced with permission.^[^ ^9b^ ^]^ Copyright 2017, Elsevier Ltd.

To realize realistic tissue morphogenesis and physiological functionality, the feasible approach is to apply a variety of cell types in a specific tissue as well as a range of cell numbers (10^4^–10^6^ or beyond) to native ECM components and obtain the tissue‐specific cell constructs. So, cell types from each solid organ must confer to strikingly different biochemical and biophysical cues to achieve tissue‐specific modeling strategies. For instance, the brain and the bone are defined by a set of completely different material properties, ECM proteins, cell types, and architectures. The ECM component contains a myriad of signaling cues, including biochemical cues from growth factors, cytokines, and adhesion ligands, and mechanical cues from ECM architecture, cell‐driven ECM forces, and surface topology.^[^ ^137^ ^]^ Generally, the ECM components in specific tissue type can provide proper microenvironment that simulates conditions in vivo. Designer peptide hydrogels are a diverse type of soft and bioactive hydrogels and assemble into a 3D nanofibrous architecture to elicit tissue‐specific signaling cues, which shows more inherent advantages than other biomaterials to mimic native ECM proteins through their spontaneous assembly (fibrillarization) and deterministic disassembly (proteolytic degradation).^[^ ^137^ ^]^ By customizing ECM‐like designer peptide hydrogels, some novel cell constructs are fabricated, which show some notable advantages over improving physiological relevance for tissue‐specific modeling compared with other hydrogels.^[^ ^132^, ^138^ ^]^ As described in **Figure** **3**, there are many types of self‐assembling peptide hydrogels to form diverse cell constructs for tissue‐specific modeling. This type of designer peptide hydrogel is capable of forming cell constructs of various 3D shapes from a wide variety of cell types at physiologically relevant cell densities and with the ability to precisely assemble and integrate different cell types in close proximity to spatial cell–cell contact in 3D context.

To achieve the physiological cell microenvironment of native tissues at largest extent, the key leap is cell density for cell phenotype keeping and tissue morphogenesis, cell development and matrix remodeling, tissue homeostasis and wound healing.^[^ ^139^ ^]^ Scaling up cell density in cell constructs is first and foremost attempt to achieve tissue organoids at sizeable amounts of cells. This approach still relies on development of a biomimetic tissue‐specific biomaterial scaffolding system that can effectively recapitulate the host microenvironment to form sizeable cell constructs and regenerate the structural and functional tissues. According to bottom‐up tissue engineering strategies, hydrogel scaffold biomaterials provide a physiochemical set of ways to control biological, mechanical, compositional, structural cues, and guide to regenerate well‐defined instructive cell constructs, which maintain the in vivo‐like homeostasis and integrative cell–ECM architecture in a biomimicry cell microenvironments in vitro.^[^ ^7^, ^132^ ^]^ By modulating the physiochemical and biological cues, the designer peptide nanofiber scaffolds can help to maintain the native cell niches or coax the encapsulated cells to form ex vivo biomimicry microtissue cell constructs in vitro.^[^ ^7^, ^21^, ^131^, ^140^ ^]^ As to amenable maneuverability in laboratory, among current scaffold biomaterials available, collagen I and Matrigel are more popular than other types of hydrogels, which are widely utilized to serve as gold standard for 3D cell culture in various cell types.^[^ ^134^, ^140^ ^]^ For clinical implantable applications, collagen I and Matrigel have largest limitations that are widely appreciated in tissue engineering and regenerative medicine, including batch‐to‐batch variability, xenogeneic sources, and incapacity to tailor the composition at nanometer scale. It is well appreciated that usefulness of Matrigel in human being is unlikely due to its nondefined composition and carcinogenicity.

For the usefulness of structurally and functionally biomimetic scaffolds, the scaffold components not only provide structural anchorage for cells but also integrate local signaling and intrinsic microarchitecture in 3D context, such as cell mobility, viable proliferation, differentiation, and phenotype dormancy. Among the increasing number of reports on implantable cell constructs, studies using natural biomaterials are rare.^[^ ^141^ ^]^ To achieve end‐product development and clinical application or to meet the contradictory requirements for commercial approval, designer self‐assembling peptide hydrogels have good manufacturing practice (GMP) considerations to reconstruct bioengineering cell microenvironments in vitro for cell constructs formation, since their molecular motifs, mesh size, nanofiber porosity, biodegradability, flexible adaptive properties favor tissue‐specific cell growth for various cell types in body, in addition to various stem cells and cancer cells.^[^ ^9^, ^10^ ^]^ As illustrated in Figure 3, designer self‐assembling peptide hydrogels emulate the physiological relevance of native ECM in vivo and serve as the biomimetic hydrogels for a broad range of tissue‐specific cell constructs appropriate for in situ critical‐sized grafting or the injury site. This type of designer peptide hydrogel is capable of forming cell constructs in a variety of adaptive shapes and inherent microarchitecture, which can be easily parallelized to produce large numbers of cell constructs in a short period. Additionally, designer self‐assembling peptide hydrogels have the flexible control to produce both homogeneous multicellular composition as well as heterogeneous ones by the precisely defined cell location. For instance, by controlling peptide backbone length and composition in amino acid residues, designer self‐assembling peptide hydrogels can be manufactured to be photosensitive or enzymatically responsive hydrogels to maintain biomimetic cell growth in vitro.^[^ ^142^ ^]^ Moreover, the size and cell density of instructive cell construct are approaching that of human beings by bioengineering nanotechnology, which promotes the translational possibilities from preclinical research to industrial development, such as the expected commercial products described above.

As described above, designer self‐assembling peptide hydrogels represent the most heterogeneous subset of hydrogels and show excellent regenerative potential and biocompatible features as fillers or scaffolds for tissue‐specific cell constructs formation in a broad range of tissues or organs. The tailor‐made peptide scaffolds may confer the transplanted cell constructs with critical size and proper cell density to ingrowth of surrounding regenerative tissues. In recent three decades, a lot of designer self‐assembling peptide hydrogels perform clinical trials in human beings.^[^ ^59^, ^60^, ^62^ ^]^ As the biomimetic cell constructs are approved in medicinal products, the advantages and benefits of designer self‐assembling peptide hydrogels are clear for the translation to a clinical platform setting, which open the ways to high‐quality clinical trials to explore the usefulness of cell construct‐based therapy by biomedical nanotechnology.^[^ ^49^, ^66^ ^]^ So, the main efforts can be put into the researches involved in complex human diseases, such as prostate cancer,^[^ ^114b^ ^]^ breast cancer.^[^ ^143^ ^]^ Herein, we focus on the several main scientific aspects involved in precise oncology remodeling of ovarian cancer and delineate the potential frontiers for future researches.

### 5.2 Precise Oncology Remodeling for Ovarian Cancer

To our knowledges, on the way of scientific progress, the history of cancer models has spanned over 100 years long, from cell monolayer culture, primary tumor transplantation in mice, establishment of stable cancer cell lines, and primary tumor histoculture on substrates to the large range of cell constructs for 3D cell cultures and tumor organoid.^[^ ^2^, ^10^ ^]^ Utilizing the advance of hydrogel matrix in regenerative medicine and bioengineering techniques, various types of cancer cells by 3D cell cultures form well‐integrated functional cell construct and closely mimic 3D cell microenvironments to explore tumorigenesis, cell growth, cell differentiation, and phenotype dormancy,^[^ ^132^, ^144^ ^]^ which recapitulate in vivo‐like cell–cell communications and cell–ECM interactions in 3D context.

#### 5.2.1 Ovarian Cancer Types and Current Cell Models

**Figure 4:**
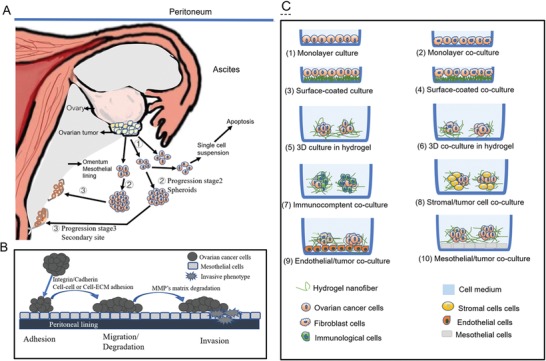
Tumor progression, peritoneal dissemination, cancer cell behaviors in ovarian cancer and schematic cell culture patterning for precise oncology remodeling in vitro. A) Schematic multistep tumor progression and peritoneal dissemination in the abdominal cavity, ➀ Single EOC cells from primary ovarian cancer site on ovary surface; ➁The primary cancer cells aggregate to survive as tumor spheroids in the ascites‐rich TMEs; ➂Partially malignant cells metastasize to the secondary site and interact with mesothelial cells to form new metastasis on the omentum. B) A schematic diagram of early‐stage primary cancer cell behaviors within a normal epithelium with breach of the peritoneal lining that separate it from the underlying stromal tissue, such as adhesion mediated by integrin/cadherin, migration promoted by extracellular solutes (growth factors, cytokines, and chemokines), and invasion induced by enzyme degradation. C) Schematic cell patterning culture models in vitro. The cartoon diagram displays the main in vitro 2D and 3D culture or coculture models, that are used to study intercellular and cell‐TME crosstalk. Two or three types of cells are incorporated to the schematic spherical 3D coculture cell models or other cell patterns.

Ovarian cancer is the ninth most prevalent cancer and the fifth leading cause of cancer‐related death in all gynecological tumors.^[^ ^145^ ^]^ Epithelial ovarian cancer (EOC) presumably accounts for ≈90% of ovarian cancer. According to genetic characteristics and different histopathology, there are at least five tumor subtypes of EOC (HGSOC, endometrioid, clear cell, mucinous, and low‐grade serous carcinomas).^[^ ^146^ ^]^ Each subtype has its own metastatic cascade, molecular aberrations and EOC cells, including ovarian cancer stem cells.^[^ ^146^, ^147^ ^]^ Of these, HGSOC is the most aggressive and common subtype, also is the most main player (75% of EOC) in the tumorigenesis, metastasis, and acquired chemoresistance in clinic medicine.^[^ ^146^, ^148^ ^]^ Since controlling peritoneal recurrence can improve survival outcomes in patients,^[^ ^146^ ^]^ **Figure** **4** depicts the scenario of intraperitoneal dissemination and cell spread in the body. Except of peritoneal lining, the omentum, a big fat pad in the peritoneal cavity, is thought to be more preferable dissemination site in ovarian cancer,^[^ ^146^, ^149^ ^]^ where omental adipocytes and fibroblasts take part in extensive desmoplastic stromal reaction. Ascites are source of early dissemination by way of transcoelomic metastasis, which contain the complex heterogeneous components consisting of single cells and multicellular aggregates or cell spheroids, stromal cells, immune cells, fibroblasts, myeloid cells, inflammatory cells, and mesothelial cells.^[^ ^147^ ^]^ So, on the onset of disease, ovarian cancer is thought to be an immunologically inert or “cold” tumor.^[^ ^150^ ^]^ The heterogeneity in cell types is one critical question for relapse and recurrence or chemoresistance occurrence in clinic treatment. It is an urgent task to develop more predictive or more comprehensive models to mimic cellular heterogeneous cues in vivo, especially complex coculture cell models with multiple cell types, that are schematically illustrated on panel C in Figure 4. In the last three decades, 5 year survival rate of ovarian cancer patients at ≈30–40% has not improved significantly, which mainly contributes to the enormous challenges in developing in vitro and ex vivo experimental cell models and raveling the potential cell mechanisms of early dissemination and genomic reprogramming progression in vivo.^[^ ^14^, ^151^ ^]^ Many reviews and reports have deeply addressed this issue.^[^ ^146^, ^147^, ^148^, ^149^, ^150^, ^151^, ^152^ ^]^

#### 5.2.2 Tumor Microenvironments

Except of multiple cell types in ovarian cancer remodeling, one distinguished feature between ovarian cancer and other kinds of human tumors is the unique TMEs in vivo,^[^ ^151c^ ^]^ where omental tissue shows extensive matrix remodeling of ECM components, with increased deposition of glycoproteins (such as fibrinogen and fibronectin) and densely packed collagen fibers. Tumor cell behaviors, such as survival, proliferation, growth, migration and invasion, are deeply dependent on the physicochemical cues of ECM components. It is evident that the ECM‐related TMEs take the center stage in tumorigenesis, progression, peritoneal dissemination, and contact metastasis.^[^ ^153^ ^]^ So, it is another urgent task to explore the advanced cell matrices to closely mimic the ECM components in TMEs of ovarian cancer, since cell functionality is often recruited to TMEs in vivo. It is ongoing task to design matrix scaffold shapes and biochemical composition to form nanoscopic and macroscopic matrices with highly tunable properties for precise oncology remodeling in ovarian cancer. Recently, inherently biodegradable scaffolds are used to examine ECM‐related signaling pathways in ovarian cancer at the molecular and cellular levels.^[^ ^152a^ ^]^ Owing to the chemical versatility of scaffold building blocks, synthetic biomaterials provide a highly comprehensive tools to create defined cell microenvironments and at largest extent maintain native tumor morphogenesis in 3D cell cultures.^[^ ^78^, ^154^ ^]^ Interestingly, designer peptide hydrogels are the upfront representatives in synthetic biomaterials available.

#### 5.2.3 Tumor Progression and Tumor Organoid

**Figure 5:**
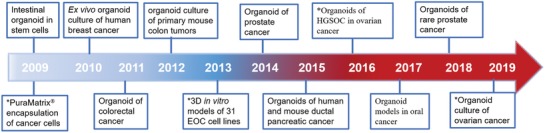
A timeline showing the development of tumor organoids. The intestinal stem cells,^[^ ^1b^ ^]^ OVCAR‐5 ovarian cancer cells,^[^ ^200^ ^]^ breast cancer progenitor cells,^[^ ^219^ ^]^ colorectal cancer cells,^[^ ^1a^ ^]^ primary intestinal cancer cells,^[^ ^220^ ^]^ EOC cell lines,^[^ ^188b^ ^]^ advanced prostate cancer cells,^[^ ^221^ ^]^ ductal pancreatic cancer cells,^[^ ^161^ ^]^ high‐grade serious ovarian cancer cells,^[^ ^222^ ^]^ oral cancer stem cells,^[^ ^157a^ ^]^ rare prostate cancer cells,^[^ ^160^ ^]^ and primary EOC cells^[^ ^162^ ^]^ represent the prominent cancer types enclosed in this review. The main experimental landmarks involved in ovarian cancer remodeling are denoted by star marks (^*^).

In 3D cancer cell cultures, it is an active research area to develop realistic and well‐defined ECM alternatives to dissect the multistep tumor progression in vivo and form in vitro microtissue constructs derived from the patients' own cells.^[^ ^4^, ^155^ ^]^ We may precisely manipulate cancer cells and matrices in vitro by the user‐directed manners and better mimic the physiologically in vivo‐like TMEs in ovarian cancer. For example, in multistep cell models of 34 cancer cell lines derived from 23 HGSOC patients to develop new treatments for therapy, it is demonstrated that loss of TP53 wild type is the main driving force of tumorigenesis and tumor progression in HGSOC.^[^ ^156^ ^]^ Cancer cell line models can be applied for clinical therapeutic targets in multistep tumor progression. Over the last decade, to develop groundbreaking therapeutic agents and recapitulate cell heterogeneity in vivo, in many tumor types, such as oral cancer,^[^ ^157^ ^]^ breast cancer,^[^ ^158^ ^]^ colorectal cancer,^[^ ^159^ ^]^ prostate cancer,^[^ ^160^ ^]^ pancreatic cancer,^[^ ^161^ ^]^ and more recently, ovarian cancer,^[^ ^162^ ^]^ 3D primary cell models in vitro have made stride forward in building tumor organoids (**Figure** **5**), a type of precise oncology remodeling, which at largest extent recapitulates the tumorigenesis in vivo. Figure 5 presents major events in the timeline of tumor organoids in the last decade. Tumor organoid is an original tool currently available to tackle a panoply of challenges in cancer biology, drug discovery, and clinical treatments, including preventing pathological tissue remodeling in vitro. Owing to tremendous advents in advanced hydrogels^[^ ^4^, ^163^ ^]^ and bottom‐up multiscale assembly in regenerative medicine and tissue engineering,^[^ ^164^ ^]^ we propose several main scientific aspects for precise oncology remodeling in ovarian cancer, such as ovarian cancer cell behaviors, exosome and acquired chemoresistance, cell–cell coculture and cell–ECM interactions, and tumor spheroid formation. Based on significance and advantages of synthetic designer peptide nanofiber scaffolds, we intend to address these main scientific aspects involved in novel 3D cell cultures in ovarian cancer. Frankly, we mainly proclaim the experimental findings and clinical reality in precise oncology remodeling in vitro and current exciting progresses in basic cancer research.

### 5.3 Precise Scientific Aspects for Ovarian Cancer Remodeling

#### 5.3.1 Ovarian Cancer Cell Behaviors

In cancer, cellular behaviors are always coupled with cell phenotypes and tumor progression in vivo. In physiological and pathological conditions, the ECM, a 3D complex network supporting the cells, is dynamically remodeled in response to various factors, including soluble or insoluble cues. A hallmark of ECM changes associated with tumorigenesis in vivo is stiffening.^[^ ^165^ ^]^ Generally, the insoluble cues alone are insufficient to illicit a full cell response, which often direct cell behaviors synergistically with the soluble biochemical factors in vivo. Accompanied with the advances in biochemistry and synthetic chemistry,^[^ ^57^ ^]^ some useful ways are developed to elucidate how soluble cues in combination with synthetic ECM induce cell behaviors in 3D context.

Chemical modification of the ECM surrounding living cells is an important means for directing cellular behaviors. In 3D cell models, a controllable method to modulate cell behaviors is to modulate biochemical and biophysical properties of synthetic ECM in user‐directed manner.^[^ ^166^ ^]^ Previously, flat substrate‐based 2D cell models enable the automated and high throughput cell assay performance in vitro, which cannot provide useful tools to analyze cell behaviors in spatial cell microenvironments. Recently, in synthetic ECM, some new techniques are described for the patterning of cell behaviors guidance cues in 3D cell models,^[^ ^167^ ^]^ where cell differentiation or cell behaviors are controlled by the synthetic hydrogels with the defined guidance cues in a biomimetic fashion. It is a helpful bioengineering methodology to customize the synthetic hydrogels to develop 3D cell models. In biomaterials area, various synthetic hydrogels are developed to investigate the cancer cell behaviors, including viable proliferation, growth, adhesion, migration, invasion, in addition to cell mechanisms involved in genomic instability and acquired chemoresistance.^[^ ^56^, ^57^, ^168^ ^]^ In order to navigate stem cell's behaviors, designer self‐assembling peptide hydrogels are applied to stem cell sorting, confined chondrogenesis and functional neural regeneration in 3D context.^[^ ^9a^ ^]^ For example, to develop serum‐free nanostructures and control cell attachment and behaviors in vitro, designer (LDLK)_3_ peptides are used to form a type of multifunctionalized hydrogels, which can support hNSCs for viable proliferation, differentiation and maturation to form the functional neuronal networks in vitro,^[^ ^67^ ^]^ where synthetic designer peptide hydrogel improves the nanofiber biomaterial–host tissue interactions after implantation and promotes cell behavioral recovery of predifferentiated hNSCs and finally form useful hNSC engraftment for hNSC therapies in vivo.

Owing to enormous molecular complexity and heterogeneity of ECM in the cellular adhesive pocket, it is very difficult to identify the specific cellular adhesive epitopes responsible for motile and invasive behaviors of cancer cells. Most recently, Carlos and co‐workers perform the cancer cell behavior assays in designer RADA16‐I peptide hydrogel.^[^ ^169^ ^]^ An easy and reliable methodology is established to decipher cancer cell behaviors and explore tumor progression in 3D context, which is also widely applicable to any type of cells, including any type of functional cell, embryonic or adult stem cells, or eventually, dysfunctional cells isolated from biopsies. As an instance, 3D cell cocultures in RADA16‐I peptide hydrogel maintain the expanded human articular chondrocytes with good viability during 4 weeks period. Due to nanoscale mesh architecture and the high water content in RADA16‐I peptide hydrogel (≈10 nm diameter and 50–200 nm pore size, a thousand times smaller than common cells), one can manipulate scaffold mesh alignment, viscoelasticity, and spatial swelling size either by tethering functional motifs or nanofiber scaffold itself. The noncovalent force interactions in nanofiber networks permit cancer cells to avoid inherent limitation for growth, migration, invasion, which confer the alteration in cell morphology and guide the proper exposition of cell surface receptors. Our studies demonstrate that 3D in vitro tumor models in designer peptide hydrogels facilitate us to explore the effects of a plethora of tumor‐promoting soluble factors or insoluble cues on cell growth, proliferation, migration, invasion, therapeutic resistance, and tumor spheroid formation.^[^ ^170^ ^]^ In common advantages of designer peptide hydrogels, tumor cells may distribute homogenously and form an intricately orchestrated signals exchange in spatial cell microenvironments, which may effectively influence cell behaviors, such as protruding cell growth.^[^ ^103^ ^]^ Moreover, peptide backbone functionalized by relevantly biological recognition and signal motifs can regulate the physiological cell behaviors in all hydrogel volume.^[^ ^169^, ^171^ ^]^ In comparison with other synthetic nanofiber hydrogels to incorporate nanofibrous architectures with spatially controllable biochemical features,^[^ ^172^ ^]^ synthetic designer peptide hydrogels are ideal reductionist mimics of the ECMs for cell behavior studies, which may create the proper topography, biophysical and biochemical cell microenvironments with physiologically relevant elasticity and viscoelasticity to direct cell behaviors.^[^ ^17^, ^109^ ^]^

Except of designer peptide hydrogels described above, some bioengineering synthetic polymer hydrogels are developed to fabricate the regenerative microenvironments in the personalized human microtissue models in vitro. The reader may refer to outside literatures for details,^[^ ^6^, ^7^, ^8^, ^18^ ^]^ Exactly, in 3D context, adhesion‐mediated signaling is affected not only by the chemical and biophysical nature of substrate scaffolds but also by the topographical features in substrate scaffolds, including the spatial patterning and alignment of the adhesive epitopes available for cell binding, the mechanical cues in the rigidity or stiffness.^[^ ^57^, ^134^, ^143^ ^]^ In 3D TMEs in vivo, where malignant cells reach protective niches in the body, cell motility and adhesion are apparently critical steps in invasive metastatic processes.^[^ ^173^ ^]^ Each cell objectively requires optimal chemical, biological, and mechanical growth conditions to modulate cell behaviors in 3D context. A series of microfluidic 3D models or 3D perfusion systems are developed to accurately recapitulate the cell microenvironments and transcoelomic routes and control the effects of soluble cues and different cell types on cell adhesion, migration, and invasiveness.^[^ ^174^ ^]^ Generally, the microfluidic platforms are fully controlled in a user‐directed manner. Both the fluidic forces and extensive soluble factors are straightforward modulators of cell behaviors in a biomimetic niche. In ovarian cancer, ascites are composed of a complex reservoir of cellular components and extracellular solutes (growth factors, cytokines, and chemokines), which nurture the shed primary tumor cells to migrate or spread to distant sites, such as peritoneum and omentum. So, cell adhesion and mobility are key in the development of 3D cell models for precise oncology remodeling in vitro. A microporous scaffold with affinity‐bound growth factors is correlated with follicular development. The follicles formed in vitro reach antral size and secreted hormones at levels leading to restoration of ovarian function.^[^ ^154^ ^]^ Largely due to technical limitations, the adhesion‐mediated signaling processes are still poorly characterized in ovarian cancer.^[^ ^150^ ^]^ Based on the advances in bioengineering cell models in cancer biology, stem cell biology, and regenerative medicine, designer self‐assembling peptide hydrogels can increasingly cater to practical 3D cell‐patterning technologies in a relevantly physiological cell culture manner.^[^ ^9^, ^10^, ^33^, ^64^, ^175^ ^]^ They may be manufactured to serve as bulk hydrogels, except of the microarchitecture similar to stromal ECM in vivo. Interestingly, designer peptide nanofiber hydrogels may independently control adhesive binding site density and matrix stiffness to influence cancer cell behaviors.^[^ ^64^, ^107^ ^]^ So, these designer peptide hydrogels can modulate the spatiotemporal cell behaviors and cell mobility of stromal cells and cancer cells or cancer stem cells in 3D context.

#### 5.3.2 Exosome and Acquired Chemoresistance

Ovarian cancer is still an easily recurrent disease. The acquired chemoresistance is one of the major challenges in clinical treatment. In patient's peritoneal cavity, exosomes in ascites are predictive of metastasis and advanced malignancy and associated with acquired chemoresistance by altering the functionality and phenotype of the recipient cells.^[^ ^176^ ^]^ In the patients with ovarian cancer, to switch to the angiogenic phenotype, VEGF levels in the ascites exceed control by 200 folds.^[^ ^177^ ^]^ Exosomes secreted by cancer cells and bystander cells in vivo can transfer a variety of molecules to manipulate TMEs so as to make it more favorable for cancer progression, such as cell phenotype dormancy, chemoresistance development, neovascularization, and distant metastasis.^[^ ^146^, ^178^ ^]^ Exosomes may regulate cellular chemoresistance at many aspects, such as controlling cell's production of key proteins and modulating the expression of MDR transporter,^[^ ^179^ ^]^ coagulation cascade,^[^ ^180^ ^]^ and intercellular signaling communication.^[^ ^181^ ^]^ To this end, the important next steps are the extensive preclinical studies using clinically representative cancer models. In a multicenter trials of 894 samples performed by the cohorts study,^[^ ^178b^ ^]^ 35 miRNAs are identified to predict early relapse or progression of epithelial ovarian cancer, that provide usefully clinic cell models in the development of a clinical‐grade prognostic assay. Except of cancer cells, macrophages‐derived exosome microRNAs contribute to the development of chemoresistance in the hypoxic TME of EOC.^[^ ^182^ ^]^ Hypoxic EOC cells trigger macrophages recruitment and induce macrophages to develop a tumor‐associated macrophage (TAM)‐like phenotype. Exosomes derived from hypoxic macrophages enhance the resistant phenotype of EOC cells. miR‐223 is enriched in this type of exosomes and can be transferred to the cocultivated EOC cells to enhance the chemoresistance via the PTEN‐PI3K/AKT pathway both in vivo and in vitro conditions. So far, the molecular composition, biological functionality, and cellular sources of exosomes have been extensively studied in ovarian cancer by 3D cell cultures.

Due to tremendous interest in translational cancer research and the basic biology, it is an enormous task to develop robust 3D cell culture models in vitro to explore the function, size, origin, morphology, and release mode of exosomes. For example, utilizing poly(2‐hydroxyethylmethacrylate) (poly‐HEMA)‐coated method to form small spheroids culture, hMSCs in vitro can produce more exosomes than 2D culture.^[^ ^183^ ^]^ The 3D stemness nano‐microenvironments on nanoculture plates are developed to obtain the massive production of exosomes,^[^ ^184^ ^]^ which enable cancer cells to form stem cell tumoroids and remodel more accurately the tumor status in morphology and gene expression in vivo. To develop the clinical translation of exosome technology, on microcarrier‐based 3D culture (commonly used scalable culture of adherent cells), Khvorova and co‐workers^[^ ^185^ ^]^ design the large‐scale method to isolate and manufacture exosomes from stem cells and yield 20‐fold more exosomes than 2D cultures. The cells are homogenously spread on microcarriers. 3D culture‐produced exosomes are seven times more potent than 2D culture‐produced exosomes. RADA16‐I peptide modified with QHREDGS, an prosurvival peptide derived from angiopoeitin‐1, is a novel delivery system of exosome‐mediated miR‐21 for progenitor stem cell transplantation,^[^ ^85^ ^]^ which reduce scar size and cell apoptosis and improve cardiac function. Due to the nonimmunogenic nature of designer peptide amphiphile, cardiac protective peptides (GHRPS) and matrix metalloprotease‐2 (MMP‐2) sequence (GTAGLIGQ) are incorporated into a designer C16 peptide to form the functionalized peptide hydrogels,^[^ ^186^ ^]^ which maintain a stable and sustained release of exosomes. So, chemically synthetic designer peptide hydrogels can increase the stability of exosomes and effectively prevent from H_2_O_2_‐induced oxidative stress. Designer peptide hydrogel can be used as an effective platform for exosome production and delivery in vitro, since they have completely known chemical compositions and convenient experimental protocols for 3D cell cultures, which facilitate the works in laboratory to elucidate the extracellular secretion, transport, quantification, and comparison of exosomes in 3D context. Although exosomes are extensively studied in cancer biology, stem cell‐based therapy, and regenerative medicines,^[^ ^187^ ^]^ based on the prominent advantages and inherent properties of designer self‐assembling peptide hydrogels, the findings described above represent the major roadblocks for the preclinical utility in translational prospects of exosomes in 3D cell cultures, which gives some basic ideas and principles in the precise oncology remodeling in vitro and exosome‐related chemoresistance mechanisms in ovarian cancer by 3D cell cultures.

#### 5.3.3 Cell–Cell Cocultures and Cell–ECM Interactions

In science community, one always pursue a robust and efficient culture system to retain clinical usefulness, genomic landscape, histopathology, and cell phenotypes of the original tumors in patients.^[^ ^188^ ^]^ Ovarian cancer is particularly relevant to the abdominal peritoneum, omentum, and ascites fluid, which simultaneously retain detached tumor cells, multiple types of stromal cells, activated mesothelial cells, fibroblasts, adipocytes, various innate, and adaptive immune cells,^[^ ^146^, ^189^ ^]^ which composes of a malicious liaison where nontumorigenic host cells interacted with various cancer cells and ECM components. In peritoneal dissemination, ovarian cancer cells are first disseminated to omentum, a mesothelium‐lined surface, which represent an initial step in transcoelomic metastasis. Second, immune cells preferably adhere to the peritoneum to localize inflammation or regulate the fluidic exchange in the peritoneal cavity. Last, omental adipocytes and fibroblasts take part in extensive desmoplastic stromal reaction and form exosomes and tissue lysates. So, to dissect the cell–cell and cell–ECM interactions in vivo, current ovarian cancer models have to develop multiple cell components coculture models,^[^ ^152^ ^]^ mainly utilizing different cell subtypes in EOC and host cells to examine cell‐cell and cell–ECM interactions in molecular and cellular levels. Since tumor spheroids are addressed in the next section, this part mainly focuses on cell patterns in 3D cocultures and cell–ECM interaction in 3D context.

In the transparent poly (l‐lactic acid) scaffolds with controlled pore size, geometry and surface properties, cancer‐associated fibroblasts (CAF), endothelial cells, macrophages and cancer cells form the multifaceted cell–cell and cell–ECM interactions in complex tissue‐engineering tumor models,^[^ ^190^ ^]^ which show spatial and quantitative aspects as similarly observed in patient‐derived peritoneal metastases. The biomimetic synthetic scaffolds provide an advanced technology platform for both in vitro and in vivo experiments of the peritoneal metastasis and facilitate cancer cells and other cells to crosstalk with their niche via tissue‐specific pathways. In ovarian cancer, the cancer‐associated fibroblast (CAF) may secrete cytokines and oncogenic ECM, a kind of metastasis‐associated stroma, which has a highly conserved functionality to drive the metabolic regulation and early tumorigenesis in vivo.^[^ ^191^ ^]^ To decipher the impact of 3D coculture on ovarian tumor microenvironment, the medical grade polycaprolactone (PCL) scaffolds are used to develop clinically predictive models seeded by primary human fibroblasts, mesothelial cells, and ovarian cancer cells,^[^ ^136b^ ^]^ which may apply to molecular stratified design of clinical trial models in the early steps of peritoneal dissemination. So, multiple cell components coculture model is one useful tool to explore the role of the TMEs (cellular and acellular) in early ovarian cancer dissemination.

As ovarian cancer rarely spreads hematogenously at early stages, cancer cells have the capacity for immunoediting to avoid the possible immune attack.^[^ ^153^, ^192^ ^]^ 3D coculture cell models are preferably used to study the cancerous immune responses and to reveal that how matrix compositions affect the reciprocal interactions between host cells and cancer cells. Compared with traditional 2D monolayer culture, the gene expression profiles of mesothelioma spheroids reveal that 112 upregulated genes are related to immune response, wound response, lymphocyte stimulation, and cytokine stimulation.^[^ ^193^ ^]^ To develop more robust 3D coculture cell models, the microfluidic device may effectively change the local cellular, molecular, chemical, and biophysical parameters in a user‐controlled manner.^[^ ^174c^ ^]^ Human macrophages are cocultured with MDA‐MB‐435S melanoma cells, MDA‐MB‐231 breast cells or PC3 prostate cells in dense collagen I. The cytokines secreted by macrophages increase the directed migration, spread, and dispersion of cancer cells and exert proximity‐induced, contact‐dependent dissemination of cancer cells. Except of physiological biophysical properties of ECM, immune cells are infiltrated into the ECM to direct cancer cell behaviors by different speed and persistence. So, except of cell–cell interactions, the matrix ECM components provide the upfront supports for immune cell growth and fate maintenance, and promoted immune cells to differentiate into the desiring phenotypes involved in each step of the metastatic cascade.^[^ ^194^ ^]^ In 3D coculture cell models, comprehensive milieu of tissue signaling may integrate the crosstalk involved in the ECMs, cancer cells, stromal cells, and immune cells in dynamic manner in 3D context. To mimic precise oncology remodeling in ovarian cancer, 3D cell–cell coculture models are the critical need to recapitulate the disease processes in vivo. Owing to the diverse cell types in ovarian cancer, as indicated in Figure 4, the robust cell–cell co‐cultures may enclose almost all cell types in the diseases and at largest extent retain the genomic, histopathological, and normal or aberrant molecular and cellular features in original tumors in vivo.

As early tissue sites in peritoneal dissemination, ovarian cancer metastasis to the omentum is more common (80%) than any other sites.^[^ ^195^ ^]^ The components of mesothelin are associated with tumor progression, cell spread, and distant metastasis. To faithfully reproduce human ovarian cancer in vivo, in 3D organotypic cell culture models, cell–ECM interactions may recreate tumor progression step by step in vitro.^[^ ^196^ ^]^ By the different ECM components in vitro, the inherent role of cell–ECM interactions are identified in the transformation of the initiating cancer cells into inert, metastatic, and resistant cancer cells. However, synthetic ECM components available restrain the organotypic models to achieve the primary tissue constructs, including access to tissue heterogeneity and the lifespan of the 3D cultures. Loessner and co‐workers use a synthetic hydrogel matrix equipped with biomimetic RGD peptides to remodel OvCa cells–ECM interactions and analyze drug resistance mechanisms in 3D context,^[^ ^197^ ^]^ which shows the flexible cell patterning manipulation and long‐term stability in 3D cultures (28 d). In our previous study, PuraMatrix hydrogel‐based 3D cell models are constructed to model the interactions between ovarian cancer cells and peptide nanofiber scaffolds.^[^ ^170^, ^199^ ^]^ SKOV3, A2780, and A2780/DDP cell lines present different cell growth patterns, such as cell colonies, cell clusters, and cell aggregates, in which peptide nanofiber scaffolds provide the biochemical and biophysical guidance cues for cell growth, viable proliferation, phenotype maintenance in hydrogel for ≈7 d. Designer peptide hydrogels are stable for cell–ECM interactions and permit long‐term experiments to be performed in vitro. Additionally, 3D cell cultures in peptide hydrogels allow us to evaluate the chemotherapeutic response of anticancer drugs at desired time points.^[^ ^143^, ^170^ ^]^ The chemosensitivity levels are quantitatively examined, since the ovarian cancer cell–nanofiber scaffold interactions in PuraMatrix hydrogel are more preferable for the chemotherapeutic responses to anticancer agents, which are also evidenced in h9e peptide nanofiber hydrogels.^[^ ^103^ ^]^ The peptide nanofiber scaffolds contain some inherent bioactive ligands and signals to enable proteolytic matrix remodeling in vitro in 3D cell culture models. The biological and mechanical properties of peptide nanofiber matrix are analogous to ECM in vivo to form proper cell–cell and cell–ECM interactions at the nanometer scale. So, designer self‐assembling peptide hydrogels have many prominent ECM features,^[^ ^78^, ^170^ ^]^ such as biocompatible, reproducible, bulk, and modular capacities. We suppose that designer peptide hydrogels are superior to the naturally derived biomaterials and synthetic polymer nanomaterials, which is an advanced or precise hydrogel matrix to mimic the native ECM cues and explore cell–ECM interactions in 3D context in vitro.

So, one proper hydrogel matrix is one useful tool to develop an efficient and robust 3D cell culture model, which facilitates to dissect cell–ECM interactions in vivo. Compared to the well‐studied hydrogels, such as Matrigel and collagen I, designer peptide scaffolds align or pattern the cell attachment in a clear and defined architecture. The cell distribution is spatially evenly and the proper physiological cell–ECM interactions are inherently formed in all hydrogel volume. These remodeling process in vitro already is envisaged in chondrogenesis, osteogenesis, and neural regeneration.^[^ ^9^, ^67^, ^199^ ^]^ For organotypic tumor models involved in MDA‐MB‐435S breast cancer cells, lumen formation is only observed in RADA16 peptide hydrogel and not in Matrigel,^[^ ^143^ ^]^ which is similar with the report of OVCAR‐5 ovarian cancer cells.^[^ ^200^ ^]^ OVCAR‐5 cells assemble into 3D acinar structures that closely resemble the morphology of micrometastatic nodules observed in clinic. So, RADA16‐I peptide hydrogel promote the tissue polarity formation in 3D cell cultures, a type of acini‐like structures, in addition to uniform and polarized nuclei, well‐organized filamentous actin, and high expression of β‐catenin, even to form normal breast acini, which evidence the cell plasticity in nanofiber hydrogel matrix. Compared to collagen I and Matrigel, the remarkable finding is that 3D cell culture of MDA‐MB‐435S cells on RADA16‐I peptide scaffolds lead to the activation of NF‐κB signaling and upregulate the intercellular surface adhesion molecule‐1 (ICAM‐1) expression and induce the stemness phenotype reversion.^[^ ^143^ ^]^ In another 3D cell culture in PuraMatrix hydrogel, the compact cell–ECM interactions are characterized at nanometer scale.^[^ ^198^ ^]^ Generally, naturally derived hydrogels often provide the hostile ECM niche for cell growth and inherently limit the direct cell–cell or proper cell–ECM interactions that are essential for early malignant progression or hinder the cell plasticity in 3D context. In contrast, N‐cadherin mimetic peptide hydrogels may be properly patterned to mimic cell–cell in vivo and native cell–ECM interactions to regulate chondrogenesis and cartilage matrix deposition.^[^ ^201^ ^]^ So, the functionality of hydrogel matrix is an important pathway to form native cell–cell cocultures and active cell–ECM interactions. The cell viability in vitro is precisely and controllably enhanced in 3D context in a user‐directed manner.

#### 5.3.4 Tumor Spheroid Formation

Multicellular tumor spheroid is often served as cancer model for precise oncology remodeling in vitro. Generally, tumor spheroid is produced by tumor cells alone or in combination with other cell types with or without matrix scaffolds.^[^ ^202^ ^]^ In shapes, tumor spheroid is round, mass, grape‐like cell aggregates, and stellate cell stacking.^[^ ^202^ ^]^ In applications, tumor spheroids are simple, accessible, and versatile cell models in vitro. Various tumor spheroid cultures are utilized to evaluate cellular functionality for preclinical drug discovery,^[^ ^76^, ^203^ ^]^ since they form closely compact tumor cell aggregates, one more relevantly physiological 3D cell models in vitro. In ovarian cancer, due to rarely involving in the vasculature,^[^ ^204^ ^]^ both scaffold‐free tumor spheroids and scaffold‐based tumor spheroid models are more preferable 3D cell models than their 2D counterparts.

In initial EOC dissemination stage, the primary tumor‐derived cells form spontaneously spheroid‐like growth and multicellular cell aggregates in peritoneal fluid or ascites, which are the hallmarks of malignant phenotype to indicate the tumorigenesis in vivo. It is evidenced that the success rate of tumor spheroid culture was only13% in HGSOC tissues,^[^ ^205^ ^]^ although ascites or peritoneal fluids generally contain fewer stromal cells and are easily dissociated. To explore patient‐specific precision medicine, it is an ongoing need to develop scaffold‐based tumor spheroid models, since scaffold‐free tumor spheroids are not susceptible to control in vitro and standardization. Herein, based on synthetic peptide hydrogel matrices, we tend to discuss scaffold‐based tumor spheroid for precise oncology remodeling in vitro in ovarian cancer.

**Figure 6:**
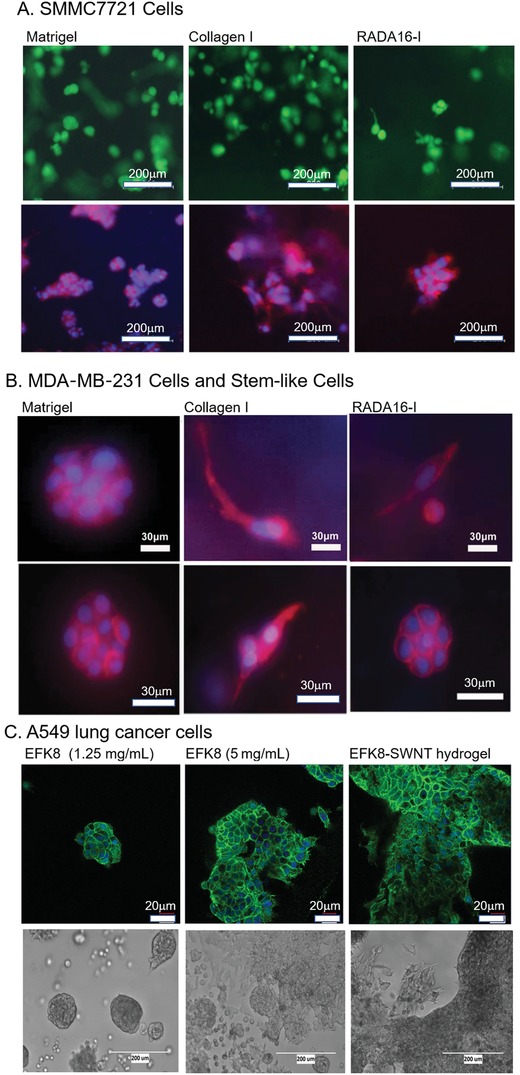
Tumor spheroid formation in several types of cancer cells in Matrigel, collagen I, and designer peptide hydrogels.^[^ ^142^, ^170^, ^223^ ^]^ A) SMMC7721 cells. B) MDA‐MB‐231S stem‐like cancer cells (reproduced with permission, copyright 2015, Dove Press); In Matrigel, both SMMC7721 cells and MDA‐MB‐231S stem‐like cancer cells form regular tumor spheroids when cultured for 3 d or 5 d (left panels). In collagen I, both SMMC7721 cells and MDA‐MB‐231S stem‐like cancer cells cannot form regular tumor spheroid growth pattern, but grow in a stretched cell shapes (middle panels); In RADA16‐I peptide hydrogel, except of MDA‐MB‐231 cells, both SMMC7721 cells and MDA‐MB‐231S stem‐like cells form regular tumor spheroid growth pattern (right panels). C) A546 lung cancer cells; when seeded on different EFK8 hydrogels, tumor spheroidal colony growth pattern is formed in 1.25 mg mL^−1^ EFK8 peptide hydrogel, 5.0 mg mL^−1^ EFK8 peptide hydrogel, and 1.25 mg mL^−1^ EFK8‐SWNT hybrid hydrogel (adapted with permission.^[^ ^196^ ^]^ Copyright 2018, Elsevier Ltd).

In early peritoneal dissemination of ovarian cancer, the dormant tumor cells always interact with the relative static ECM components in vivo, which mainly consist of a nanofibrous mesh of structural proteins such as fibronectin, collagen, elastin, and laminin.^[^ ^35^, ^132^ ^]^ These ECM components are key to support various cell signaling communications and cell phenotype transformation and maintain tumor cells survival in vivo and the malignant proliferation. The enormous efforts are performed to mimic the ECM components in vivo and fabricate various functional hydrogel matrix for 3D tumor spheroid cultures.^[^ ^14a^ ^]^ As to synthetic hydrogels, integrin‐binding motifs or other adhesive sites are often incorporated to mimic the native ECMs at largest extent. For instance, Loessner and co‐workers^[^ ^197^ ^]^ prepare a type of RGD‐functionalized hydrogel matrices to produce bioengineering tumor spheroids in ovarian cancer and compare spheroid growth, intraperitoneal spread, and chemoresistance in malignant progression. RGD motifs for integrin activation drive the large spheroid formation by the viable cell proliferation in 3D context. In synthetic peptide hydrogels, the defined chemical composition and the active functionality are the prominent features to form useful and enough large tumor spheroids in vitro. This type of synthetic peptide hydrogels not only easily support 3D cell organization but also introduce cell guidance cues to porous nanofiber scaffolds, that intimately mediate cell–cell adhesion and cell–matrix interactions in a user‐directed manner. In designer self‐assembling peptide hydrogels, each scaffold may provide specific biochemical and mechanical features for tumor spheroid formation. In some tumor types other than ovarian cancer, prostate cancer is predominant alternative option for tumor spheroid formation in vitro.^[^ ^35b^ ^]^ The stem‐like prostate cancer cells are embedded in designer peptide hydrogels and remain quiescent status for more than 28 d.^[^ ^206^ ^]^ The cell viability and stem‐like property are maintained consistently in vitro. Q11, bQ13, and RADA16‐I peptide hydrogels are used to culture LNCaP prostate cancer cells^[^ ^114b^ ^]^ and produce a broad range of tumor spheroids with an unpolarized, disorganized cell aggregates, that are greatly analogous to Matrigel and superior to collagen I. So, designer self‐assembling peptides are ideal candidates to reconstruct the conducive cell microenvironment and to maintain cell survival and physiological functionality by tumor spheroids, which may achieve organotypic tumor spheroid constructs in vitro.^[^ ^207^ ^]^ In 3D cell culture in Matrigel, collagen I and RADA16‐I peptide hydrogels,^[^ ^170b^ ^]^ SMMC7721 cells show cell aggregates growth pattern and the nanofiber networks of hydrogel matrix affect cell construct morphology in 3D context (**Figure** **6**A). When compared with collagen I and Matrigel,^[^ ^143^ ^]^ tumor cells in designer peptide hydrogels maintain the polarized cell colonies in the cell aggregates state for 3 weeks (Figure 6B). OVCAR‐5 ovarian cancer cells^[^ ^200^ ^]^ are cultured in RADA16‐I peptide hydrogel. On days 3, multicellular tumor spheroids are formed. On days 7, OVCAR‐5 cells form big tumor spheroids with a size of 100 µm and a transparent overview. The hybrid self‐assembling peptide (EFK8)‐carbon nanotube (SWNT) hydrogels are designed to observe A549 lung cancer cell morphology from spheroidal to a stretched alteration similar to migratory phenotype in malignant solid tumors (Figure 6C).^[^ ^105^ ^]^ A thin layer of RADA16‐I peptide hydrogel may support a miniature ovarian cancer cell pattern for drug chemosensitivity detection when seeded with low ovarian cancer cell density.^[^ ^198^ ^]^ So far, by tumor spheroid culture in 3D context, the clonal diversity, cell adaptation, drug repositioning, and malignant phenotype reversion are envisioned in various designer peptide hydrogels described above. We suppose that designer peptide hydrogels are superior to naturally derived hydrogels and other synthetic hydrogels for precise oncology remodeling in vitro.

Tumor spheroid size typically ranges from 100 to 700 µm in diameter.^[^ ^203a^ ^]^ There is a fundamental issue to produce large enough tumor spheroids with the controllable size of cell aggregates and stable diffusion of oxygen, nutrients and waste in 3D cell cultures in vitro. In dynamic 3D cell culture models, the fluid shear stress improves the roundness and size consistency of tumor spheroids.^[^ ^203a^ ^]^ In a 384 well hanging drop array platform,^[^ ^208^ ^]^ the stable and uniform tumor spheroids are produced by as few as 10 cells incorporated into a single initial culture, which provides a powerful system on high throughput 384 well plates to explore ovarian cancer spheroid biology in rare patient‐derived cells. It is evident that the dynamic cultures need some special equipment and technology. A synthetic hydrogel microwells array of poly(ethylene glycol)(PEG)‐based hydrogel is designed to generate tumor spheroids with a reproducible and homogenous size.^[^ ^209^ ^]^ Chemically synthetic hydrogels have widely been used for microwell fabrication, which may incorporate multiple cell types to generate well‐defined and complex 3D cell–cell coculture models. This nonadhesive hydrogel is used to develop uniform size microwell arrays for high‐throughput drug screening in vitro. To retain both histological and genetic features and intratumor heterogeneity in the tumor spheroids in vivo, adhesive property‐based synthetic scaffold is an alternative option to generate self‐assembled 3D cellular tumor spheroids in static cell cultures, such as chitosan and HA.^[^ ^210^ ^]^ Although the molecular structures and biological properties are similar with the polysaccharide components of ECM, there are few reports to address the feasible experimental protocols to generate uniform tumor spheroids. In bulk manufacture production of tumor spheroids in vitro, designer self‐assembling peptide hydrogels may directly print the viable tumor spheroid constructs by 3D bioprinting techniques without physical or chemical postprocessing.^[^ ^4b^ ^]^ Moreover, designer peptide hydrogels may be customized to the biomimetic nanostructures amenable of active functionalization with the superior biocompatibility to other synthetic hydrogels.^[^ ^76^, ^211^ ^]^ Designer peptide scaffolds have high‐porosity and resemble the arrangement of ECM components in vivo at the nanometer scale, which support different levels of cell density and high cell viability when embedded in hydrogel matrices. The tumor spheroids formed allow the high cell plasticity and extensive matrix remodeling corresponding to the organotypic reconstruction way towards the scenario in vivo. Compared with other synthetic hydrogels, another benefit is easy recovery of cells in tumor spheroid formation in vitro, which is demonstrated by experimental assays.^[^ ^103^ ^]^ From the point of view in translational applications, utilizing designer peptide hydrogels to produce tumor spheroid with uniform size is no doubt one useful tool in basic cancer research and clinical or preclinical drug discovery.

## 6 Conclusions and Outlook for Future

Designer self‐assembling peptides are an advanced type of biomimetic nanofiber matrices, which open up the avenues to engineer cell microenvironments in regenerative medicine, tissue engineering, basic cancer research, biomedical nanotechnology, preclinical drug development, and clinical drug discovery. Compared with naturally derived hydrogels and synthetic or semisynthetic hydrogels, chemically synthetic designer peptide hydrogels have diverse building block types, which provide a large range of biomimetic nanofiber matrix alternatives to form highly organized cell constructs with high cell density and active intercellular signaling communication. For tissue‐specific cell constructs formation, the reciprocal entanglement of peptide nanofiber networks through noncovalent interactions are greatly attractive for in situ gelation to integrate multiple cell types and ECM components with the spatial and temporal control in a user‐directed manner.

The future trend still is the manipulation of the physiological complexity in 3D cell culture assays, which includes not only different cell types, but also an increase of options in matrix composition and functionality as well as the inclusion of the temporal and spatial presentation of soluble or insoluble cues in all hydrogel volumes. Recent remarkable advances in designer self‐assembling peptide hydrogels appreciate us to discuss the bioengineering TMEs in various tumor cell models in vitro, due to the inherent biocompatibility, potential biodegradability, tunable viscoelasticity, flexible manufacture manipulation, and similar nanometer scale with ECM components in vivo.

Accompanied with the underlying genomic events driving ovarian cancer progression,^[^ ^162^ ^]^ it is appreciated that ovarian cancer is as molecularly distinct entity as breast, prostate, and renal cancer.^[^ ^212^ ^]^ The comparative molecular studies that fail to recognize the histopathological origins are no longer effective.^[^ ^150^, ^213^ ^]^ Novel ovarian cancer cell models are ongoing need to reflect the main scientific aspects for precise oncology remodeling in vitro, such as cancer cell behaviors, exosomes, and acquired chemoresistance, cell–cell coculture and cell–ECM interactions, and tumor spheroids formation, which elaborately recapitulate cell–cell and cell–ECM interactions in ovarian cancer development, progression, metastasis, dissemination or therapeutic recurrence and relapse. To capture the oncogenic drivers and tumor biology of HGSOC, fallopian tube‐based model systems may define the physiology and susceptibility of this epithelium to tumorous transformation.^[^ ^214^ ^]^ So, much attention must be strictly paid to the human ovarian cancer cell models to explore different aspects in the tumorigenesis. In the future, as the avenues toward personalized precise medicine take a leap to explore more and more precise 3D cell models in vitro for patient‐specific therapies and wide patient‐derived cancer research,^[^ ^144a^ ^]^ immense efforts should be taken into account to explore growth conditions of particular subpopulations of tumor cells and high density coculture systems with immune cells or other cell subtypes.

To achieve pathological and physiological complexity in vivo, the availability of cell models in vitro is a prerequisite for successful preclinical and translational researches in current cancer biology. It is timely to consider the precise oncology remodeling in vitro on the verge of these major challenges that propel subtype‐specific maintenance therapy and alongside increasingly sensitive disease detection tools in clinic medicine. Particularly, by patient‐derived primary tumor cells, tumor organoids represent currently innovative tools to moving complex tumor progression and clinical drug development into the age of precision medicine.

## Conflict of Interest

The authors declare no conflict of interest.
